# Metal Cluster Size-Dependent Activation Energies of Growth of Single-Chirality Single-Walled Carbon Nanotubes inside Metallocene-Filled Single-Walled Carbon Nanotubes

**DOI:** 10.3390/nano11102649

**Published:** 2021-10-09

**Authors:** Marianna V. Kharlamova, Christian Kramberger

**Affiliations:** 1 Institute of Materials Chemistry, Vienna University of Technology, Getreidemarkt 9/BC/2, 1060 Vienna, Austria; 2 Moscow Institute of Physics and Technology, Institutskii Pereulok, 9, 141700 Dolgoprudny, Russia; 3 Faculty of Physics, University of Vienna, Boltzmanngasse 5, 1090 Vienna, Austria; Christian.Krambegrer-Kaplan@univie.ac.at

**Keywords:** single-walled carbon nanotube, double-walled carbon nanotubes, growth kinetics, growth mechanism, growth rate, activation energy, Raman spectroscopy

## Abstract

By combining in situ annealing and Raman spectroscopy measurements, the growth dynamics of nine individual-chirality inner tubes (8,8), (12,3), (13,1), (9,6), (10,4), (11,2), (11,1), (9,3) and (9,2) with diameters from ~0.8 to 1.1 nm are monitored using a time resolution of several minutes. The growth mechanism of inner tubes implies two successive stages of the growth on the carburized and purely metallic catalytic particles, respectively, which are formed as a result of the thermally induced decomposition of metallocenes inside the outer SWCNTs. The activation energies of the growth on carburized Ni and Co catalytic particles amount to 1.85–2.57 eV and 1.80–2.71 eV, respectively. They decrease monotonically as the tube diameter decreases, independent of the metal type. The activation energies of the growth on purely metallic Ni and Co particles equal 1.49–1.91 eV and 0.77–1.79 eV, respectively. They increase as the tube diameter decreases. The activation energies of the growth of large-diameter tubes (*d_t_* = ~0.95–1.10 nm) on Ni catalyst are significantly larger than on Co catalyst, whereas the values of small-diameter tubes (*d_t_* = ~0.80–0.95 nm) are similar. For both metals, no dependence of the activation energies on the chirality of inner tubes is observed.

## 1. Introduction

Single-walled carbon nanotubes (SWCNTs) were discovered in 1993 [1,2] and are a fascinating class of carbon nanomaterials possessing exceptional physical, chemical, mechanical and structural properties [3]. They are unique nanoscale objects, because their electronic structure (metallic or semiconducting) is solely dependent on the atomic structure [3,4]. Since the discovery of SWCNTs, the work of many researchers has been aimed at developing methods for their efficient synthesis. In recent years, significant progress was made in this field. The arc-discharge, laser ablation and chemical vapor deposition (CVD) methods were optimized for the synthesis of SWCNTs in a high yield [5,6]. Synthesis parameters can be varied in a broad range, which leads to the production of SWCNTs with defined morphology and high purity. Although the selective synthesis of SWCNTs with certain conductivity types and structures was attempted [7,8], typical as-synthesized samples consist of a mixture of metallic and semiconducting SWCNTs [6]. This causes inhomogeneity of their properties.

The methods for the chemical functionalization of nanotubes, such as the substitution of carbon atoms of SWCNT walls, the intercalation of SWCNT bundles, the functionalization of the SWCNT outer surface and the filling of SWCNT inner channels, were developed to control the properties of nanotubes. They open up a way of tailoring the doping level of nanotubes and even switching between doping types [9].

The synthesis of SWCNTs with defined properties is required for both fundamental investigations and practical applications. Despite the fact that the use of SWCNTs in the fields of nanoelectronics [10,11,12,13], thin-film flexible electronics [14,15] and bioelectronics [16] has already been realized, the full potential of SWCNTs is still not implemented in applications. The revealing and thorough understanding of the growth mechanism of SWCNTs is the key to the synthesis of nanotubes that possess the required properties.

Growth dynamics of SWCNTs in the CVD process wasstudied [17,18,19,20,21,22,23]. However, the short synthesis time of SWCNTs due to the fast passivation of the catalytic particles impeded the precise investigation of nanotube growth and the obtainment of quantitative data. Most works were dedicated to studies on the growth of SWCNT forests and therefore could not enable information on the rates and activation energies of the growth of individual tubes to be deduced. In [21,22], the growth rates were measured for single-chirality SWCNTs. Nevertheless, in these works, the systematic investigation of the influence of synthesis parameters on growth dynamics of nanotubes is lacking.

To overcome these problems, growth dynamics of SWCNTs should be studied in a stable system where nanotube growth occurs with a slow enough rate over a sufficiently long time. Growth dynamics of nanotubes should be monitored *in situ* using a precise method that is suitable for long-term measurement and is able to provide quantitative data. The synthesis conditions of nanotubes should be well-controlled. The applied synthesis system should provide as precise as possible control over the temperature, diameter of nanotubes and the chemical composition of the catalyst and carbon feedstock.

A way to achieve this is by using metallocene-filled SWCNTs as the synthesis system. It was demonstrated that annealing filled SWCNTs leads to the formation of inner tubes, and the inner tube growth can continue for many hours [24,25]. The filled SWCNTs serve as a catalyst source, carbon feedstock and container, providing a shielded environment for the nanotube growth at the same time [24]. The thermally induced decomposition of metallocene leads to a fixed stoichiometry of metal to carbon atoms, and therefore the chemical composition of catalyst and carbon source is specified. The charge transfer and doping level of the filled nanotubes can be precisely controlled [25,26]. The diameter of the inner tubes is defined by the diameter of the outer SWCNTs, and thus it can be controlled through the choice of pristine SWCNT material.

The aim of this work is to calculate metal cluster size-dependent activation energies of growth of single-chirality inner SWCNTs inside metallocene-filled SWCNTs, to elucidate differences in the rates and activation energies of the growth of inner tubes inside nickelocene- (NiCp_2_) and cobaltocene (CoCp_2_)-filled SWCNTs and to reveal the growth mechanism of the inner tubes.

## 2. Experimental Section

### 2.1. Synthesis of Filled SWCNTs

SWCNT buckypapers were prepared *via* enhanced direct injection pyrolytic synthesis (e-DIPS), as described in [27]. The method is based on the chemical vapor deposition of the carbon source on catalytic particles that are formed in situ in the reactor. The toluene (the first carbon source) solution of the catalyst precursor, ferrocene with a concentration of 4 wt% and an equimolar amount of thiophene relative to iron atoms was used as a feedstock. The feedstock was injected directly into a vertical ceramic tube reactor under a hydrogen carrier gas flow. The latter contained ethylene gas as the second carbon source. The synthesis was conducted when the temperature of the furnace was 1200 °C [27]. According to the HRTEM and optical absorption spectroscopy data presented in Refs. [27,28], the samples of the e-DIPS SWCNTs contained nanotubes with diameters ranging from ~1.1 to 2.3 nm. Their mean diameter was 1.7 nm.

### 2.2. Preparation of SWCNT Films

To homogenize the nanotubes, films were formed from the initial buckypaper samples. The sample (m = 3 mg) was dispersed in toluene (≥99.5%, Carl Roth GmbH+Co. KG, Karlsruhe, Germany) (V = 20 mL) *via* bath ultrasonication for 1 h. This dispersion was filtered through a 4.7 cm-diameter cellulose filter membrane with a pore diameter of 0.2 µm (Millipore) using a vacuum filtration setup. The obtained membrane containing the SWCNTs was put into methanol (≥99.5%, Carl Roth GmbH+Co. KG) (V = 45 mL) and ultrasonicated for a few minutes to disperse the nanotubes. After that, the membrane was removed, and the SWCNTs were ultrasonicated in methanol for 30 min. The obtained dispersion was filtered through a 2.5 cm-diameter cellulose filter membrane with a pore diameter of 0.2 µm (Millipore). Nine milliliters of dispersion was needed for the formation of one nanotube film. As soon as the methanol was evaporated, the film was peeled off the membrane. The obtained nanotube films had a mass of about 0.6 mg and a diameter of 1 cm.

### 2.3. Opening of SWCNT Ends

Prior to being filled, the nanotube films were annealed in air at 500 °C for 1 h in order to open the SWCNT ends. The effectiveness of this procedure was confirmed in the filling experiments.

### 2.4. Filling of SWCNT Channels

The filling of SWCNTs with metallocene molecules (nickelocene ((C_5_H_5_)_2_Ni, NiCp_2_) and cobaltocene ((C_5_H_5_)_2_Co, CoCp_2_) was performed using the gas-phase method. The molecules were observed to decompose at temperatures higher than 60–70 °C. However, they were easily sublimated in vacuum at temperatures as low as 50 °C without a noticeable decomposition. Therefore, the following technique was used for the filling of the nanotubes. The pre-opened SWCNT film and metallocene powder ((C_5_H_5_)_2_Ni or (C_5_H_5_)_2_Co, 99%, Strem Chemicals Inc., Bischheim, France) were placed into a glass tube (Pyrex, Chateauroux, France). This procedure was performed in a glove box in an atmosphere of argon, because metallocenes are easily oxidized in air. Then, the tube was connected to a turbopump (Pfeiffer vacuum, Vienna, Austria) that provided vacuum better than 10^−6^ mbar and evacuated for 20 min. After that, the SWCNTs and metallocene powder were sealed into an ampoule. The ampoule was heated using a heating coil made from a wire—a Thermocoax (Paris, France) heating element with a Ni-Cr alloy core, MgO insulant and stainless steel sheath. The temperature was controlled using a laboratory power supply (Heinzinger (Rosenheim, Germany) or TDK-Lambda(Achern, Germany)). Half of the ampoule was heated up to 50 °C. This led to the sublimation of metallocene powder and its desublimation in the cold half of the ampoule. Depending on the amount of metallocene powder, this process lasted for 12–24 h. Then, the position of the ampoule was changed so that the half of the ampoule with metallocene would be heated. This procedure was repeated 5–10 times over 5 days. After the filling experiment was finished, the ampoule was opened in a glove box. The filled nanotube samples are labeled MCp_2_@SWCNT, where M = Ni, Co.

### 2.5. Formation of DWCNTs

DWCNTs were formed *via* the thermal treatment of the metallocene-filled SWCNTs. It was performed *in situ* (while the sample was located inside the setup for spectroscopic measurements) and *ex situ* (using a furnace).

#### 2.5.1. In Situ Annealing

**Figure 1:**
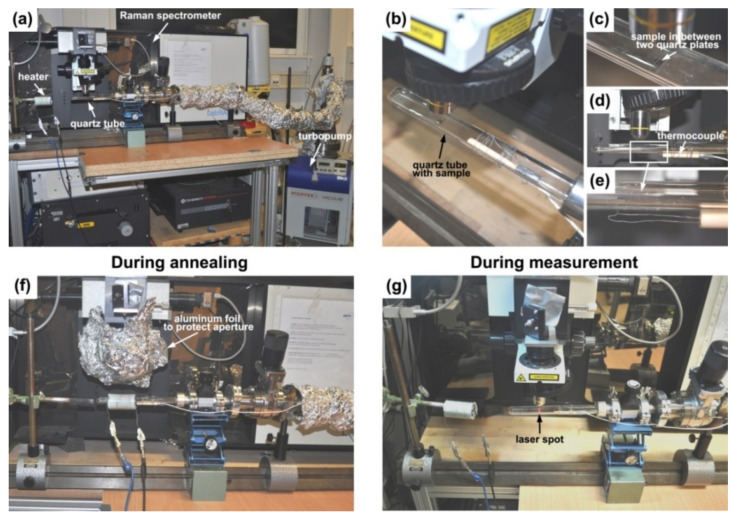
The setup used for *in situ* annealing and Raman spectroscopy measurements: (**a**) the overall view where the main parts of the setup are denoted by arrows, (**b**) the top view of the quartz tube with sample positioned under the objective of the spectrometer, (**c**) the enlarged view of the sample placed in between two quartz plates inside the quartz tube, (**d**) the side view of the quartz tube, presenting the thermocouple fixed at the bottom of the tube, (**e**) the enlarged view of the end of the thermocouple from (**d**) located below the sample, (**f**) the view of the setup during annealing, (**g**) the view of the setup during measurement.

Figure 1a shows the overall view of the setup that was used for *in situ* annealing and Raman spectroscopy measurements. The setup included a flat quartz tube (QSIL GmbH, Ilmenau, Germany) with the metallocene-filled nanotube sample connected to a turbopump (Pfeiffer vacuum) providing vacuum better than 10^−6^ mbar, which was placed near a Raman spectrometer (Horiba Jobin Yvon LabRAM HR800 (Tulln, Austria)). The annealing was performed using a home-built resistive heater that was connected to a power supply (Xantrex, Elkhart, IN, USA) with a temperature controller (Eurotherm, Limburg an der Lahn, Germany). The filled nanotube sample in the form of a film with a size of about 5 × 5 mm^2^ was placed in between two quartz plates inside a flat quartz tube, as shown in Figure 1b,c. The quartz tube was located so that the nanotube film was positioned under an objective of the spectrometer. At the bottom of the quartz tube, a home-built thermocouple (type K) made from Ni-Cr and Ni-Al alloy wires (Omega Resistance Wire, Manchester, UK) was mounted so that its end was located directly below the film, as demonstrated in Figure 1d,e. The other end of the thermocouple was connected to the temperature controller.

During annealing, the heater was placed on the quartz tube so that its middle coincided with the position of the sample and the end of the thermocouple. The objective of the spectrometer was removed, and the aperture was covered with a protective aluminum foil, as shown in Figure 1f. The *in situ* annealing was performed at a fixed temperature, step by step for doubled periods of time, excluding the time required for the heating of the system. In all experiments, the heat up time was 2 min. Therefore, the durations of the annealings were described by the formula τ= 2 + 2n, where n = 1, 2, 3,..., 12 and τ unit is minutes. Thus, the experiments were conducted over 4, 6, 10, 18, 34, 66, 130, 258, 514, 1026, 2050 and 4098 min.

The *in situ* annealings were performed at several temperatures with a step of 20 °C. Such fine temperature control was required because the growth rates of the inner nanotubes increased rapidly with a small increase in annealing temperature, such as 20 °C. A step of 20 °C was chosen as the minimal possible, taking into consideration the accuracy of the temperature determination using the thermocouple. The annealing of the nickelocene-filled SWCNTs was carried out at seven different temperatures: 480, 500, 520, 540, 560, 580 and 600 °C, and the annealing of the cobaltocene-filled nanotubes was performed at six temperatures: 540, 560, 580, 600, 620 and 640 °C.

After every annealing step was finished, the heater was removed from the quartz tube and the cooled down sample was studied using Raman spectroscopy (Figure 1g). Details on the Raman spectroscopy measurements are presented in next section.

#### 2.5.2. Instrumentation for Raman Spectroscopy

*In situ* Raman spectroscopy investigations of the metallocene-filled SWCNTs and DWCNTs were performed using a Horiba Jobin Yvon LabRAM HR800 spectrometer adapted for multifrequency measurements, as described in [29]. The system was equipped with an internal He/Ne laser operating at a wavelength of 633 nm (energy of 1.96 eV) and an external tunable Ar/Kr mixed gas laser (Coherent Innova 70c, Dieburg, Germany) operating at wavelengths of 458, 488, 514, 531, 568 and 647 nm (energies of 2.71, 2.54, 2.41, 2.34, 2.18 and 1.92 eV, respectively).

**Figure 2:**
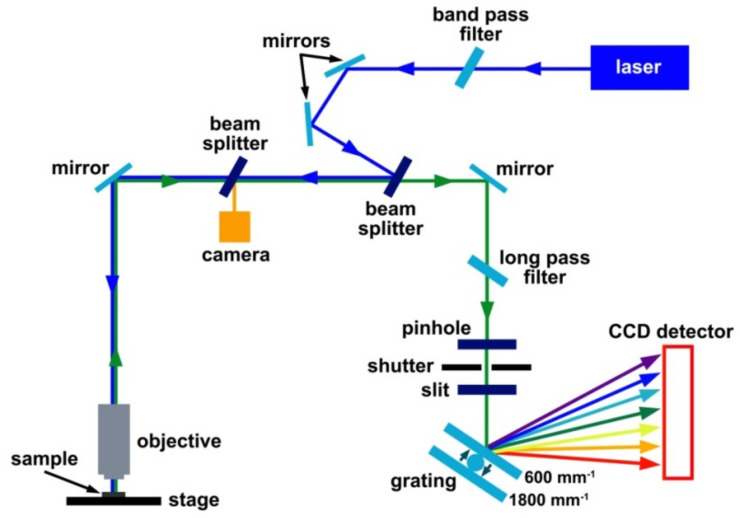
The schematics of Horiba Jobin Yvon LabRAM HR800 Raman spectroscopy system. The main parts of the setup are labeled. The incoming laser beam is shown in blue. The Raman scattered light is shown in green. The directions of the laser beams are guided by arrows.

Figure 2 demonstrates the schematics of the Raman spectroscopy system. The laser beam (shown in blue) is guided into the spectrometer through a band pass filter, *via* mirrors and several beam splitters (in transmission or reflection regime) and focused with a 50× objective on a sample positioned on a stage. Raman scattered light (shown in green) is collected by the objective and follows the same optical path as the incoming laser beam until the first beam splitter. It is transmitted by the beam splitter and guided *via* mirrors through a long pass filter, pinhole, shutter and slit to a grating, where it is dispersed by wavelength, and then recorded with a liquid nitrogen cooled charge coupled device (CCD) detector (Symphony, Paris, France). The system is equipped with a camera positioned at the beam splitter that is the nearest to the objective. When the beam splitter is in the beam path, most of the laser beam is reflected, and the sample can be inspected using the camera. When the beam splitter is out of the beam path, the laser beam follows through the objective to the sample. This is the Raman measurement mode. The measurements can be conducted in normal and high-resolution regimes, depending on the grating used. A switching between 600 and 1800 mm^−1^ gratings leads to changing from the normal to high-resolution regime. The rotation angle of the gratings relative to the laser beam defines the recorded spectral range.

*In situ* Raman spectroscopy studies were conducted at laser wavelengths of 568 and 633 nm. The choice of these laser wavelengths for the experiments was motivated by two reasons. Firstly, multifrequency Raman spectroscopy measurements showed that the peaks of the inner tubes had the maximal intensity in the Raman spectra acquired at 568 and 633 nm. Secondly, Raman spectra acquired at 568 and 633 nm contained peaks of nine distinct inner tubes with different diameters and chiralities, which allowed a comprehensive picture of the dependence of inner tube growth on their diameter and chirality to be obtained.

The measurements were performed according to the following procedure. In the camera mode, the laser beam was focused by a 50× objective on a nanotube film that was placed in between two quartz plates inside a flat quartz tube connected to a turbopump, in order to achieve the maximal intensity of the signal. Then, the system was switched to the Raman measurement mode and the spectrum acquisition was conducted with a constant incident laser power of 3.4 mW, a 1000 µm pinhole and a 100 µm slit. The complete spectral range from 50 up to 3000 cm^−1^ was recorded using a 600 mm^−1^ grating (normal resolution mode). The spectral range was divided into several windows. Every window was measured in 5 s and it was repeated 12 times in order to achieve good-quality data. The total acquisition time of the spectrum was about 7 min. After that, the radial breathing mode region from 100 to 500 cm^−1^ was recorded using an 1800 mm^−1^ grating (high resolution mode). The measurement was carried out in a single window over 15 s and it was repeated 72 times. The measurement took about 20 min. All measurements were performed at room temperature.

The nanotube films obtained after *ex situ* annealings were studied using multifrequency Raman spectroscopy at laser wavelengths of 458–647 nm using the above-described system. For the measurements, the samples were attached to a sticky aluminum foil. The spectra were recorded in the range from 50 up to 3000 cm^−1^. A constant incident laser power of 0.5 mW, a 1000 µm pinhole, a 100 µm slit and a 600 mm^−1^ grating were used. The measurement of the complete spectral range was performed in a multiwindow regime. Every window was measured over 5 s 12 times. One measurement lasted for about 7 min. Additionally, samples were investigated using a Brucker RFS 100/S FT spectrometer equipped with anNd:YAG laser with a wavelength of 1064 nm (1.17 eV). A constant incident laser power ranging between 100 and 150 mW was used. Every spectrum was acquired in the range from −1400 to 3500 cm^−1^ with 4000 scans. The spectral resolution was 1 cm^−1^. The measurement time was about 6–8 h.

The RBM-bands of the Raman spectra were fitted to Voigtian peaks, and the area intensities were calculated using PeakFit v4.12. The error in peak position was ±2 cm^−1^. For the comparison of the complete range of spectra acquired at different laser wavelengths, they were normalized to the area intensity of the G-band (between 1350 and 1700 cm^−1^), in order to exclude effects of differences in focusing.

## 3. Results

To investigate the inner tube growth, the NiCp_2_-filled SWCNTs were annealed at fixed temperatures between 480 and 600 °C step by step during periods of time ranging from 2 up to 5069 min. The CoCp_2_-filled SWCNTs were annealed at temperatures in the range from 540 to 640 °C for periods of time ranging from 2 to 4168 min. The evolution of their Raman spectra acquired at laser wavelengths of 633 nm (E_ex_ = 1.96 eV) and 568 nm (E_ex_ = 2.18 eV) was analyzed after each annealing step for every annealing temperature. Lasers with wavelengths of 568 and 633 nm were used for two reasons. Firstly, the most intense peaks of inner tubes are observed in the spectra acquired at these laser wavelengths, whereas the peak intensities are sufficiently lower in the spectra obtained with other lasers. Secondly, the diameters of inner tubes detected with these laser wavelengths are in a broad range from ~0.7 to 1.3 nm.

### 3.1. Scanning and Transmission Electron Microscopy

**Figure 3:**
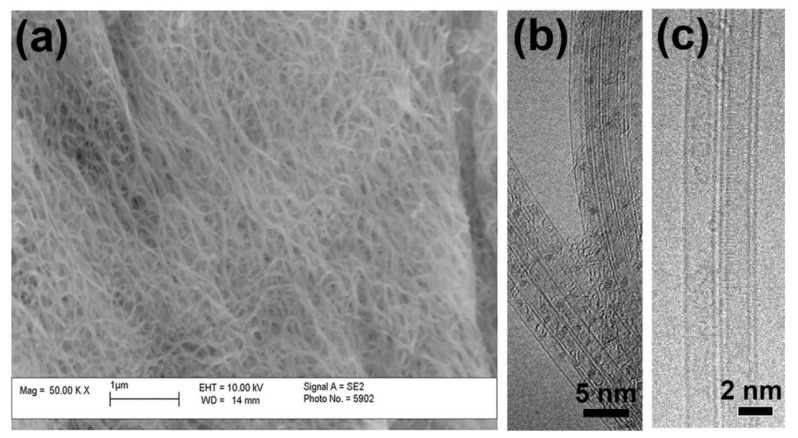
(**a**) The SEM image of the pristine SWCNTs, (**b**) the TEM image of NiCp_2_-filled SWCNTs annealed at 200 °C, (**c**) the TEM image of DWCNTs obtained by annealing of CoCp_2_-filled SWCNTs at 750 °C.

Figure 3a shows the scanning transmission electron microscopy (SEM) image of the pristine SWCNTs. It is visible that the nanotube film consists of homogenous SWCNTs. The encapsulation process leads to the filling of SWCNTs with metallocene, as shown in the transmission electron microscopy (TEM) image in Figure 3b. The channels of SWCNTs are filled. The annealing of the filled SWCNTs in vacuum results in the decomposition of metallocene with the formation of DWCNTs (Figure 3c).

### 3.2. In Situ Raman Spectroscopy at Laser Wavelengths of 633 and 568 nm

**Figure 4:**
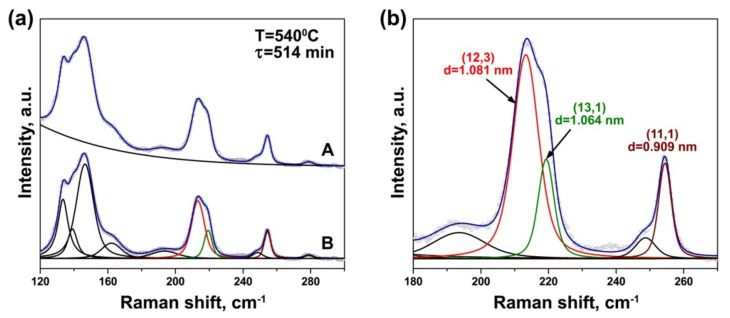
(**a**) The fitting of the RBM-band of the Raman spectrum of the NiCp_2_-filled SWCNTs annealed at 540 °C for 514 min, acquired at a laser wavelength of 633 nm (E_ex_ = 1.96 eV), with the components of individual outer and inner tubes. The spectrum with the fitted background (A) and the same spectrum with the subtracted background fitted with individual components (B). (**b**) The enlarged view of the fitting of the inner tube peaks. The most intense inner tube peaks are labeled.

The RBM-bands of the Raman spectra of the annealed NiCp_2_- and CoCp_2_-filled samples acquired at laser wavelengths of 633 and 568 nm were fitted with the components of individual outer and inner tubes. Figure 4 illustrates the fitting procedure of the spectrum acquired with the 633 nmlaser using an example of the NiCp_2_-filled sample annealed at 540 °C for 514 min. As shown in Figure 4a, the background was subtracted from the spectrum. The outer tube peak, which is composed of the overlapping peaks of different nanotubes, was fitted with four individual components positioned at 134, 139, 146 and 162 cm^−1^. They correspond to outer tubes with diameters of ~1.86, 1.78, 1.68 and 1.49 nm, respectively [30]. Taking into consideration the Kataura plot, the peaks stem from optical transitions between the third/forth van Hove singularities in semiconducting SWCNTs [31]. The most intense peak at 146 cm^−1^ belongs to the mean diameter nanotubes (~1.68 nm). The inner tube peaks were fitted with five individual components centered at 194, 214, 219, 249 and 254 cm^−1^. Figure 4b demonstrates an enlarged view of the fitting of the inner tube peaks. The three most intense components at 214, 219 and 254 cm^−1^ can be assigned to the (12,3) nanotube with a diameter of 1.081 nm, the (13,1) tube with a diameter of 1.064 nm and the (11,1) tube with a diameter of 0.909 nm, respectively [31,32,33]. The first and second components are in resonance with the first optical transition in metallic nanotubes, and the third component—with the second optical transition in semiconducting tubes [31]. Table 1 summarizes the fitting results of the inner tube peaks and the data on the assigned nanotubes.

It should be noted that the van der Waals distance between the outer and inner graphene layers of DWCNTs is 0.335 nm. Consequently, for the pristine SWCNTs with a mean diameter of 1.7 nm, the mean diameter of the formed inner tubes is ~1.0 nm. Thus, the detected (12,3) and (13,1) tubes correspond to the mean diameter inner nanotubes.

**Figure 5:**
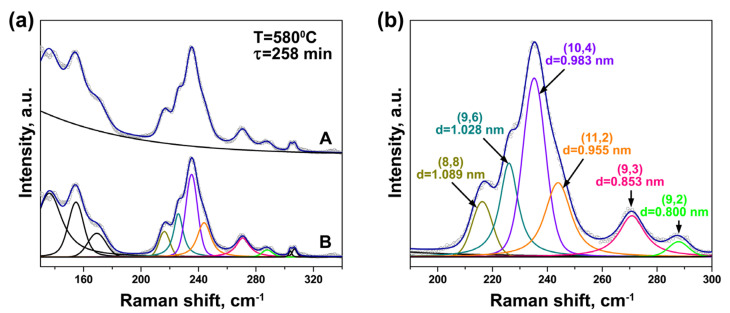
(**a**) The fitting of the RBM-band of the Raman spectrum of the CoCp_2_-filled SWCNTs annealed at 580 °C for 258 min, acquired at a laser wavelength of 568 nm (E_ex_ = 2.18 eV), with the components of individual outer and inner tubes. The spectrum with the fitted background (A) and the same spectrum with the subtracted background fitted with individual components (B). (**b**) The enlarged view of the fitting of the inner tube peaks. The inner tube peaks are labeled.

Figure 5 illustrates the fitting procedure of the spectrum acquired with a 568 nm laser using an example of the CoCp_2_-filled sample annealed at 580 °C for 258 min. After the background was subtracted from the spectrum, the outer tube peak was fitted with three components centered at 136, 155 and 169 cm^−1^. They correspond to outer tubes with diameters of ~1.82, 1.56, 1.42 nm, respectively [30]. According to the Kataura plot, the peaks originate from the third/forth optical transitions in semiconducting SWCNTs [31]. The inner tube peaks were fitted with eight individual components centered at 215, 226, 235, 240, 269, 285, 303 and 306 cm^−1^ (Figure 5a). Figure 5b demonstrates an enlarged view of the fitting of the inner tube peaks. The most intense components positioned at 215, 226, 235, 240, 269 and 285 cm^−1^ correspond to the (8,8) nanotube with a diameter of 1.089 nm, the (9,6) tube with a diameter of 1.028 nm, the (10,4) tube with a diameter of 0.983 nm, the (11,2) tube with a diameter of 0.955 nm, the (9,3) tube with a diameter of 0.853 nm and the (9,2) tube with a diameter of 0.800 nm, respectively [31,32,33]. The diameters of the detected (8,8), (9,6), (10,4) and (11,2) tubes are close to the mean diameter of the inner nanotubes (1.0 nm). The first five components stem from the first optical transition in metallic nanotubes, and the sixth component—from the second optical transition in semiconducting tubes (Table 1) [31].

**Figure 6:**
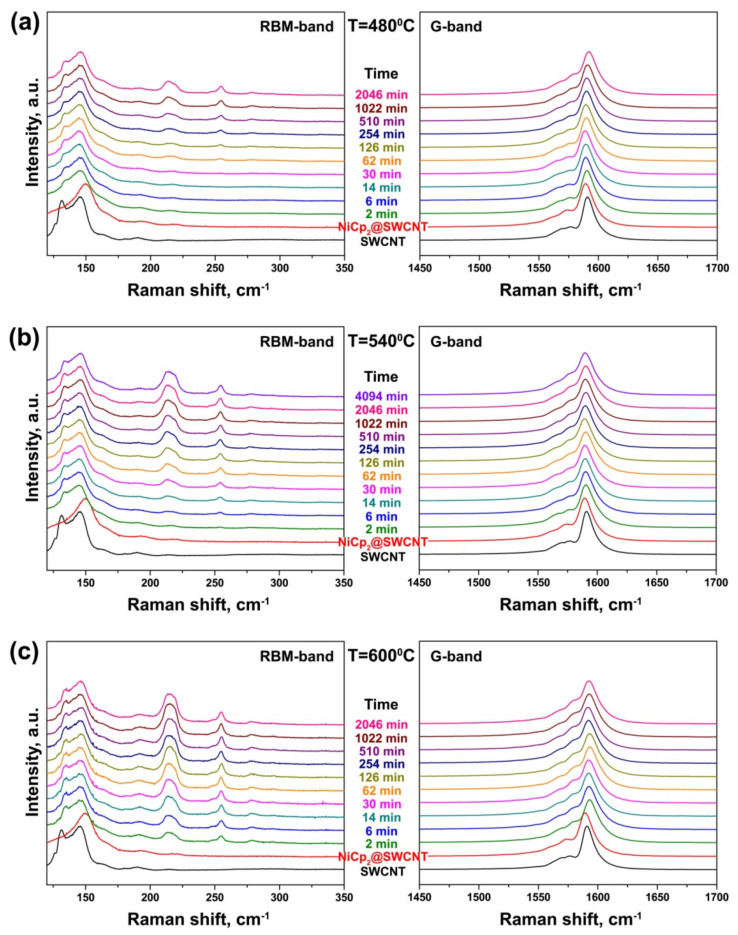
The evolution of the RBM- and G-bands of the Raman spectra of the nickelocene-filled SWCNTs upon their *in situ* annealing at temperatures of 480 °C (**a**), 540 °C (**b**) and 600 °C (**c**) for 2–4094 min. The Raman spectra are acquired at a laser wavelength of 633 nm (E_ex_ = 1.96 eV).

Figure 6 presents the evolution of the RBM- and G-bands of the Raman spectra of the nickelocene-filled SWCNTs, obtained at an excitation laser wavelength of 633 nm, upon their *in situ* annealing at 480, 540 and 600 °C for 2–4094 min. The RBM-band of the spectrum of the pristine SWCNTs demonstrates two prominent peaks at 132 and 146 cm^−1^. They correspond to the nanotubes with diameters of ~1.89 and 1.68 nm, respectively [30]. The RBM-band of the spectrum of the NiCp_2_-filled SWCNTs shows an upshift of the peak of the mean diameter tubes to 150 cm^−1^, which is common for molecule-filled SWCNTs [24]. The peak of the larger-diameter tubes disappears, which might be connected with strong deviations from tubular symmetry due to the encapsulated molecules (Figure 6).

The RBM-bands of the spectra of the annealed samples obtained at all annealing temperatures include the peak of the mean diameter of SWCNTs, which is downshifted by 4 cm^−1^ to the position of the pristine tubes. Additionally, the spectra demonstrate the recovery of the peak of the larger-diameter outer nanotubes, centered at 132 cm^−1^, which becomes more prominent with increasing annealing time and temperature (Figure 6). This might be connected with the restoration of tube symmetry due to the decomposition of the encapsulated nickelocene. Additionally, new peaks of inner tubes centered at 214, 219 and 254 cm^−1^ arise in the spectra after a certain annealing step (Figure 6). The inner tube peaks appear after different annealing durations in the spectra of the samples annealed at different temperatures. In the spectra of the samples annealed at 480 °C, the peaks become visible after 62 min of annealing (Figure 6a), and the annealing time required for the formation of inner tubes decreases gradually to 6 min at 540 °C and 2 min at 600 °C (Figure 6b,c). At all annealing temperatures, the intensity of inner tube peaks increases with increasing annealing time.. However, the maximal intensity of peaks achieved after the latest annealing steps of similar durations differs in the spectra obtained at different annealing temperatures. It increases significantly with increasing temperature. The most intense peaks of inner tubes are observed in the spectrum of the sample annealed at 600 °C for 2046 min (Figure 6c). Their large relative intensity as compared to the peak of outer tubes is indicative of the high filling degree of the SWCNTs with nickelocene molecules, resulting in the formation of DWCNTs in a large yield.

The high filling leads to a strong Raman response of inner tubes in the G-bands of Raman spectra of the annealed samples, too. All of them contain an intense peak at frequencies ranging from 1550 to 1625 cm^−1^ (Figure 6). It is centered at 1590 cm^−1^ in the spectrum of the pristine SWCNTs. The peak is slightly downshifted by 2 cm^−1^ in the spectrum of the NiCp_2_-filled SWCNTs, and it shifts back in the spectra of the annealed samples. Moreover, its shape changes significantly, because of an additional component of inner tubes. This component is placed at 1578 cm^−1^, lower than the outer tube peak by 12 cm^−1^. It appears due to phonon softening in smaller-diameter tubes [34]. The intensity of this component is enhanced with an increase in annealing time for all annealing temperatures, which leads to the broadening of the G-band (Figure 6).

**Figure 7:**
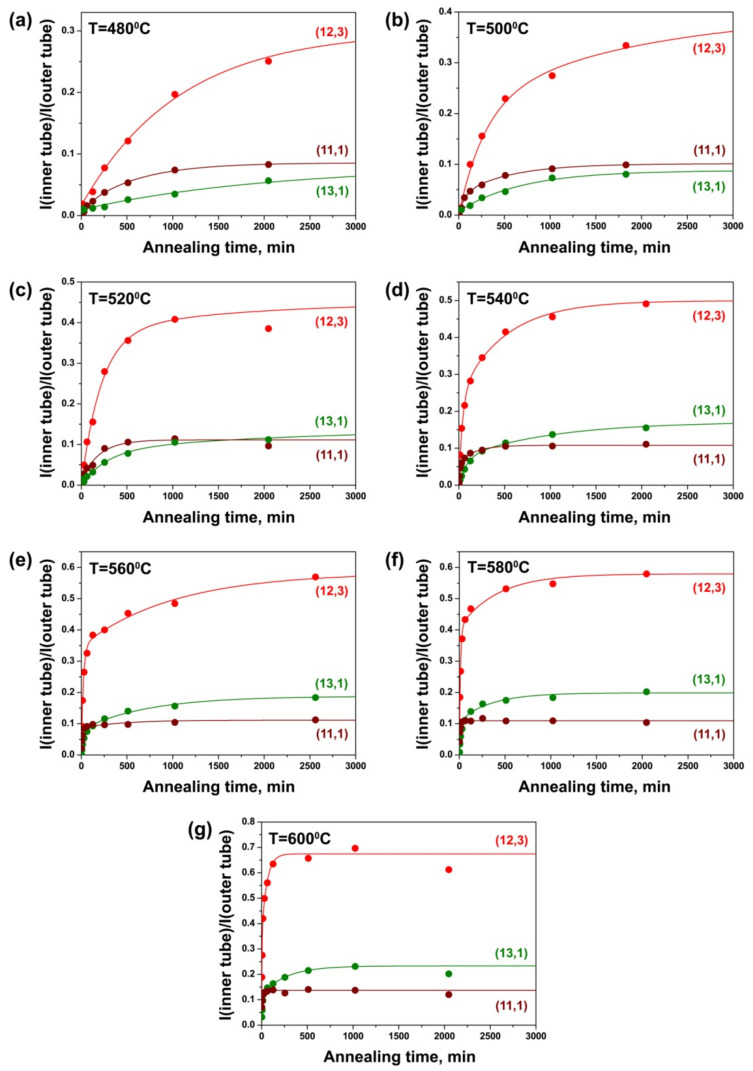
The integral intensity of peaks of the (12,3), (13,1) and (11,1) inner tubes normalized to the intensity of the outer tube peak observed in the RBM-band of Raman spectra of the annealed NiCp_2_-filled SWCNTs acquired at a laser wavelength of 633 nm (E_ex_ = 1.96 eV) plotted versus annealing time. The experimental data for the samples annealed at temperatures of 480 °C (**a**), 500 °C (**b**), 520 °C (**c**), 540 °C (**d**), 560 °C (**e**), 580 °C (**f**) and 600 °C (**g**) are shown as filled circles. The fitting results of the data are presented as solid lines.

Changes in the intensity of the components of inner tubes with increasing annealing duration were traced in the Raman spectra of the NiCp_2_- and CoCp_2_-filled annealed samples. Figure 7 shows the integral intensity of the components of the (12,3), (13,1) and (11,1) inner tubes, observed in the spectra acquired with a 633 nm-laser, normalized to the intensity of the outer tube peak plotted versus annealing time for the NiCp_2_-filled samples annealed at different temperatures. These growth curves of inner tubes demonstrate that growth dynamics is different for various nanotubes and it depends strongly on annealing temperature.

The curves obtained at 480 °C represent a slow gradual increase in the peak intensity with annealing time for all nanotubes (Figure 7a). The inner tube growth becomes faster with increasing annealing temperature. At 500 °C, the growth curves of the (12,3) and (11,1) nanotubes are characterized by a significant increase in the peak intensity at the first several steps of annealing (up to 500–1000 min) and a slower increase in the intensity at the following annealing steps. The curve of the (13,1) tube demonstrates a slow nanotube growth at all annealing steps (Figure 7b). At 520 °C, the curves of all tubes show their fast growth during the first 500 min of annealing. The curve of the (13,1) tube again demonstrates the slowest changes in the peak intensity. At further annealings, only small changes in the inner tube peak intensities are observed for the (12,3) and (13,1) tubes, whereas the intensity of the (11,1) tube peak stays constant (Figure 7c). At 540 °C, all tubes grow even faster during the first 500 min of annealing, and a slow growth continues at further annealing steps. For the (11,1) nanotube, the intensity of the peak is almost saturated after 254 min (Figure 7d). At 560 °C, the (12,3) and (13,1) tubes grow very fast during the first 62 min of annealing and much slower at the next annealing steps. However, the peak intensities are still not saturated after the last annealing (2560 min). The intensity of the peak of the (11,1) tube increases significantly during 14 min of annealing and reaches a saturated value after 62 min (Figure 7e). At 580 °C, after an initial burst for 62 min, the intensities of the (12,3) and (13,1) tube peaks increase slowly at following annealing steps and saturate after 2046 min. For the (11,1) tube, the intensity increases quickly during 14 min of annealing and is almost saturated after 30 min (Figure 7f). At 600 °C, the intensities of inner nanotube peaks grow extremely fast during the first minutes of annealing, and they are practically saturated after 510 min in the case of the (12,3) and (13,1) tubes and 30 min in the case of the (11,1) tube (Figure 7g).

Thus, all inner nanotubes grow faster with increasing annealing temperature. At all annealing temperatures, the (11,1) tube, which has the smallest diameter, was observed to grow significantly faster than the (12,3) and (13,1) nanotubes. This testifies that the growth rates of inner tubes depend on their diameter: smaller diameter tubes grow faster than larger diameter nanotubes.

**Figure 8:**
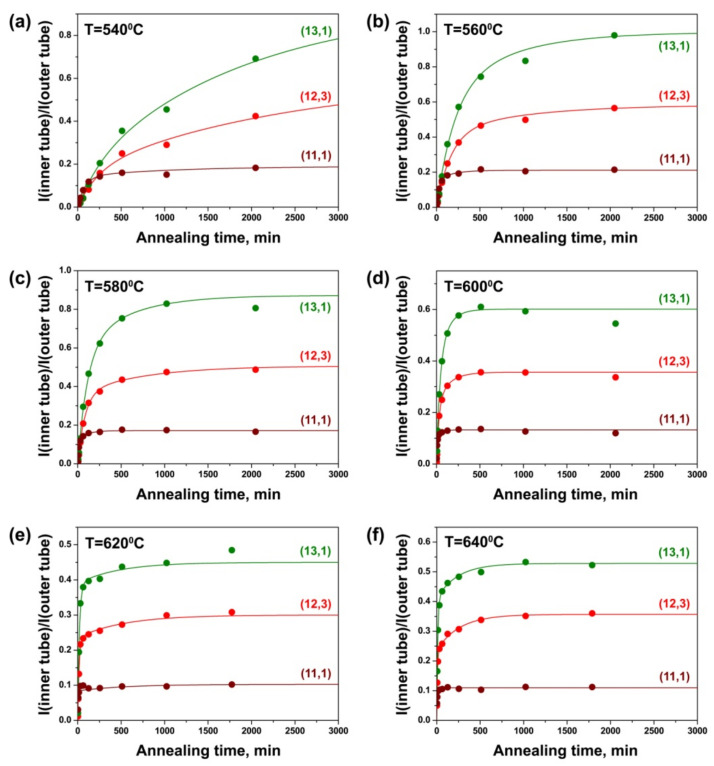
The integral intensity of peaks of the (12,3), (13,1) and (11,1) inner tubes normalized to the intensity of the outer tube peak observed in the RBM-band of Raman spectra of the annealed CoCp_2_-filled SWCNTs acquired at a laser wavelength of 633 nm (E_ex_ = 1.96 eV) plotted versus annealing time. The experimental data for the samples annealed at temperatures of 540 °C (**a**), 560 °C (**b**), 580 °C (**c**), 600 °C (**d**), 620 °C (**e**) and 640 °C (**f**) are shown as filled circles. The fitting results of the data are presented as solid lines.

Figure 8 shows the integral intensity of the components of the (12,3), (13,1) and (11,1) inner tubes normalized to the intensity of the outer tube peak plotted versus annealing time for the CoCp_2_-filled samples annealed at different temperatures. The growth curves of various inner tubes reveal noticeable differences. At the same time, the growth dynamics of inner tubes depends strongly on annealing temperature. At 540 °C, the curves of the (12,3) and (13,1) tubes demonstrate a slow gradual increase in the peak intensity with annealing time. The curve of the (11,1) tube shows a significant growth in the peak intensity during the first 254 min of annealing, only small changes in the intensity at further annealing steps and the saturation after 2046 min (Figure 8a). At 560 °C, the growth curves of the (12,3) and (13,1) nanotubes are characterized by a noticeable increase in the peak intensity during the first 510 min of annealing and slower increase in the intensity at the following annealing steps. However, the peak intensities are still not saturated after the last annealing step (2046 min). The intensity of the peak of the (11,1) tube rises significantly during the first 126 min of annealing and reaches a saturated value after 510 min (Figure 8b). At 580 °C, the (12,3) and (13,1) tubes grow quickly during the first 254 min of annealing, and their intensities are almost saturated after 2046 min. The (11,1) tube grows rapidly during the first 62 min of annealing, and the intensity of the peak is saturated after 254 min (Figure 8c).

With a further increase in annealing temperature, the periods of time of an initial burst and saturation of the peak intensity are reduced for all inner tubes. At 600 °C, the peaks of the (12,3) and (13,1) tubes are characterized by a fast growth of the intensity for 126 min and saturation after 510 min. For the (11,1) tube, the peak intensity increases rapidly during 30 min of annealing and saturates after 126 min (Figure 8d). At 620 °C, after an initial burst for 62 min, the intensities of the (12,3) and (13,1) tube peaks saturate after 510 min. The (11,1) tube grows quickly during the first 14 min, and the peak intensity is almost saturated after 62 min (Figure 8e). At 640 °C, all inner nanotubes grow even faster during the first minutes of annealing, and the intensities of the tube peaks are saturated after 510 min in the case of the (12,3) and (13,1) tubes and 62 min in the case of the (11,1) tube (Figure 8f).

Thus, the obtained data show that the growth dynamics of inner tubes does not follow a simple exponential model, as in the case of the SWCNT growth in the CVD process. After an initial fast growth for several minutes, inner tubes continue to grow for tens of hours. The (11,1) tube, possessing the smallest diameter, grows significantly faster than the (12,3) and (13,1) nanotubes at all annealing temperatures. The growth of all inner tubes is accelerated with increasing annealing temperature. The same trends were observed for the growth dynamics of inner tubes inside the nickelocene-filled SWCNTs.

While comparing the growth curves of inner tubes inside the NiCp_2_- and CoCp_2_-filled SWCNTs, one can notice differences in the relative intensities of the (12,3) and (13,1) tube peaks. In the case of the CoCp_2_-filled SWCNTs, the intensity of the peak of the (13,1) tube is larger than the one of the (12,3) tube at all annealing durations and temperatures (Figure 8). For the NiCp_2_-filled nanotubes, there is the opposite ratio of the intensities of the tube peaks (Figure 7). Taking into consideration the fact that the same pristine SWCNT sample was used for the filling with these molecules, the observed different relative intensities of the inner tube peaks can be related to the different chemical natures of nickelocene and cobaltocene. Indeed, it was reported that both molecules have a similar “sandwich” structure, with a metal ion centrally placed between two cyclopentadienyl rings [35,36,37], to ferrocene—the first discovered [38,39] member of the family of metal dicyclopentadienyl compounds [40,41,42,43]. The metal–carbon distances obtained from the experimental data (electron and X-ray diffraction) and density functional theory (DFT) calculations were reported to increase in the line with Fe-Co-Ni. The Fe-C distances were found to be in the range from 2.007 to 2.064 Å [44,45,46,47,48,49], the Co-C distances from 2.070 to 2.120 Å [37,49,50,51,52,53] and the Ni-C distances from 2.148 to 2.196 Å [36,49,54,55]. This is explained by changing the electronic structure of the molecules in the line with FeCp_2_—CoCp_2_—NiCp_2_. In FeCp_2_, 18 electrons (5 from each of the rings and 8 from the iron atom) are distributed among strongly bonding (a_1g_, a_2u_, e_1g_, e_1u_), weakly bonding (e_2g_) and nonbonding ($a_{1g}^{'}$) molecular orbitals [36,37,56]. In CoCp_2_ and NiCp_2_, an additional one and two electrons, respectively, occupy $e_{1g}^{*}$ antibonding orbitals, which leads to the weakening of the metal–carbon bonding in the molecule [36,37,56]. The increase in the metal–carbon distances results in the increase in the molecule size from ferrocene to cobaltocene to nickelocene.

The difference in the size of NiCp_2_ and CoCp_2_ molecules may lead to different filling degrees of the outer tubes of the same diameter. Taking into account the diameters of the (12,3) and (13,1) inner tubes (1.081 and 1.064 nm, respectively) and the van der Waals distance between the outer and inner graphene layers of DWCNTs (0.335 nm), the diameters of the corresponding outer tubes are estimated to be 1.751 and 1.734 nm, respectively. We assume that the outer diameter is critical for the packing density of the molecules. In the case of nickelocene, more dense packing is formed in the larger-diameter outer tube, which results in a higher yield of the larger-diameter (12,3) inner tubes as compared to the (13,1) tubes (Figure 7). In the case of the smaller cobaltocene molecule, the same dense packing is also possible for the smaller-diameter outer SWCNT, which leads to an increased yield of the smaller diameter (13,1) inner tubes (Figure 8).

**Figure 9:**
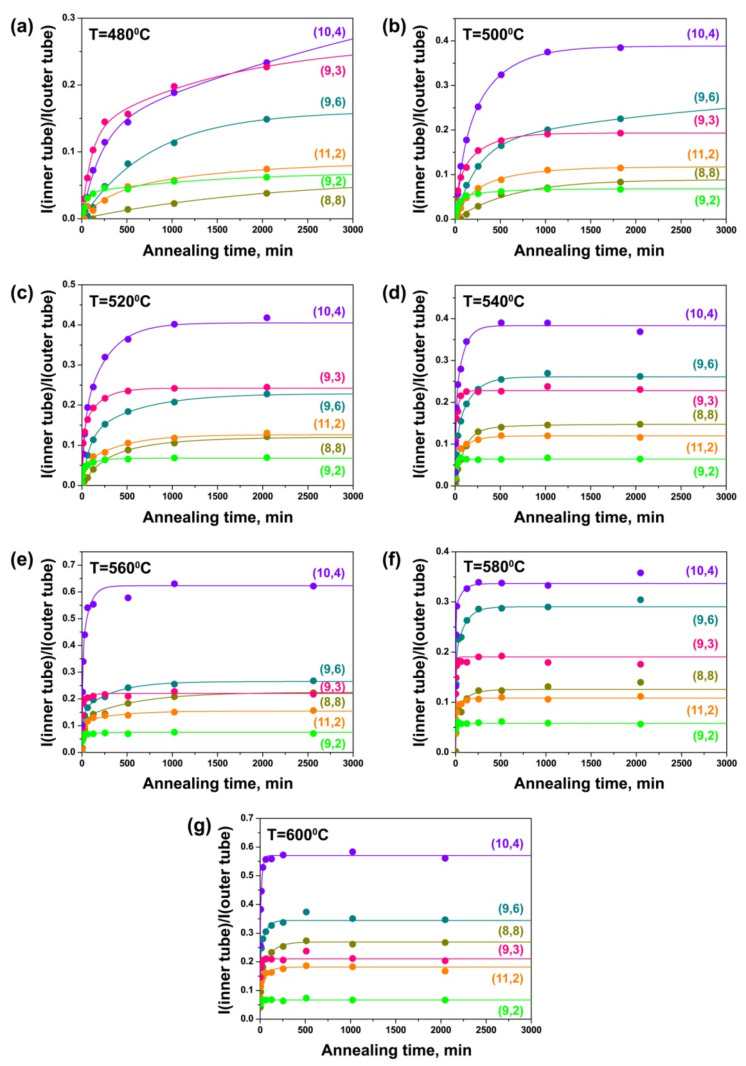
The integral intensity of peaks of the (8,8), (9,6), (10,4), (11,2), (9,3) and (9,2) inner tubes normalized to the intensity of the outer tube peak observed in the RBM-band of Raman spectra of the annealed NiCp_2_-filled SWCNTs acquired at a laser wavelength of 568 nm (E_ex_ = 2.18 eV) plotted versus annealing time. The experimental data for the samples annealed at temperatures of 480 °C (**a**), 500 °C (**b**), 520 °C (**c**), 540 °C (**d**), 560 °C (**e**), 580 °C (**f**) and 600 °C (**g**) are shown as filled circles. The fitting results of the data are presented as solid lines.

Figure 9 shows the integral intensity of the components of the (8,8), (9,6), (10,4), (11,2), (9,3) and (9,2) inner tubes, observed in the spectra acquired with a 568 nm-laser, normalized to the intensity of the outer tube peak plotted versus annealing time for the NiCp_2_-filled samples annealed at different temperatures. The growth curves of all nanotubes are significantly changed with increasing annealing temperature. At the same time, the curves of the nanotubes with different diameters show noticeable differences. At 480 °C, the curves of the (8,8), (9,6), (10,4) and (11,2) tubes demonstrate a slow growth of the peak intensity with annealing time. The (9,3) and (9,2) nanotubes with smaller diameters grow faster: their curves represent a large increase in the peak intensity during the first 254 and 126 min of annealing, respectively, and a slower increase in the intensity at the following annealing steps (Figure 9a). At 500 °C, all tubes grow quicker. Only the growth curve of the (8,8) nanotube with the largest diameter shows a gradual increase in the peak intensity with annealing time. The curves of all other tubes are characterized by a significant increase in the peak intensity at the beginning of annealing and a much slower increase in the intensity later (Figure 9b). The (9,6) tube grows rapidly during the first 510 min of annealing, and the peak intensity is not saturated after the last annealing step (1830 min). The (10,4) and (11,2) tubes grow faster during the first 510 min, and the peak intensities reach the saturated values after 1830 min. The intensity of the peak of the (9,3) tube changes rapidly during 254 min of annealing and becomes almost saturated after 1022 min. The (9,2) tube with the smallest diameter grows with the fastest rate. The significant change in the peak intensity occurs during 126 min and the value is saturated after 510 min. At 520 °C, the (8,8), (9,6), (10,4) and (11,2) tubes grow even faster during the first 510 min of annealing. The peak intensity reaches the saturated value after 2046 min for the (8,8) and (9,6) tubes and after 1022 min for the (10,4) and (11,2) tubes. The peaks of the (9,3) and (9,2) tubes increase significantly during the first 126 and 30 min, respectively, and their intensities are saturated after 510 and 254 min, respectively (Figure 9c).

With a further increase in annealing temperature, the time period of fast nanotube growth and the annealing duration required to achieve the saturated values of the peak intensities decrease. At 540 °C, the intensities of the peaks of the (8,8), (9,6), (10,4) and (11,2) tubes are saturated after 510 min of annealing and the (9,3) and (9,2) tubes—after 126 min (Figure 9d). At 560 °C, the intensities of the peaks of the (8,8) and (9,6) tubes reach maximal values after 510 min, the (10,4) and (11,2) tubes—after 254 min, the (9,3) tube—after 62 min and the (9,2) tube—after 30 min (Figure 9e). At 580 °C, the time equals 254 min for the (8,8), (9,6) and (10,4) tubes, 126 min—for the (11,2) tube, 30 min—for the (9,3) tube and 14 min—for the (9,2) tube (Figure 9f). At 600 °C, the time equals 254 min for the (8,8) tube, 126 min—for the (9,6), (10,4) and (11,2) tubes, 30 min—for the (9,3) tube and 14 min—for the (9,2) tube (Figure 9g).

According to these data, there is a clear tendency toward significant increase in the growth rates of inner tubes with increasing annealing temperature of the nickelocene-filled SWCNTs. Additionally, the growth rates of inner tubes increase with a decrease in the inner tube diameter. The (8,8) and (9,6) tubes with the largest diameters were observed to grow with the slowest rates. The (9,3) and (9,2) tubes with the smallest diameters grew with the fastest rates.

**Figure 10:**
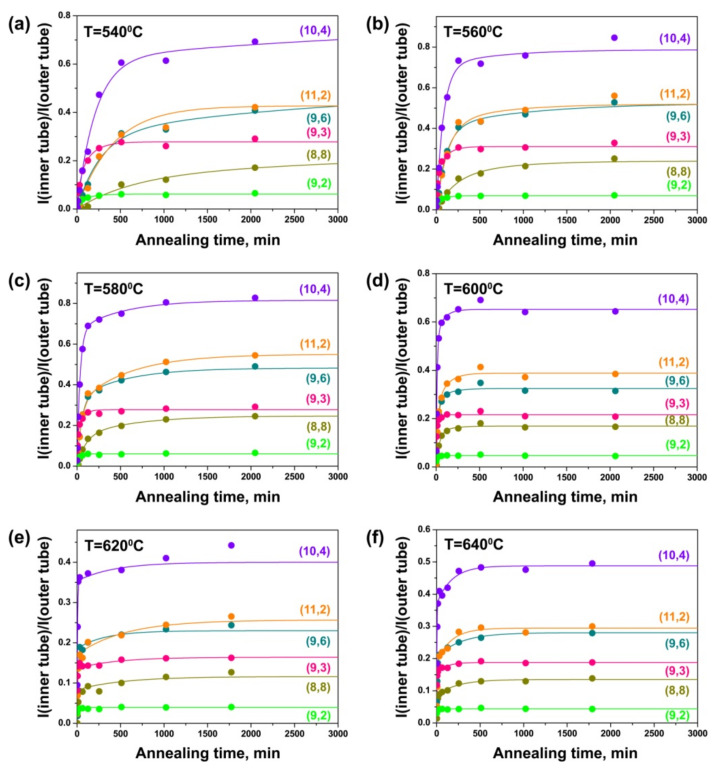
The integral intensity of peaks of the (8,8), (9,6), (10,4), (11,2), (9,3) and (9,2) inner tubes normalized to the intensity of the outer tube peak observed in the RBM-band of Raman spectra of the annealed CoCp_2_-filled SWCNTs acquired at a laser wavelength of 568 nm (E_ex_ = 2.18 eV) plotted versus annealing time. The experimental data for the samples annealed at temperatures of 540 °C (**a**), 560 °C (**b**), 580 °C (**c**), 600 °C (**d**), 620 °C (**e**) and 640 °C (**f**) are shown as filled circles. The fitting results of the data are presented as solid lines.

Figure 10 demonstrates the dependencies of the integral intensity of the components of the (8,8), (9,6), (10,4), (11,2), (9,3) and (9,2) inner tubes normalized to the intensity of the outer tube peak on annealing time for the CoCp_2_-filled samples annealed at different temperatures. At 540 °C, the growth curve of the (8,8) nanotube shows a gradual increase in the peak intensity with annealing time. The curves of all other tubes are characterized by a significant growth of the peak intensity at the beginning of annealing and a much slower growth of the intensity later. The intensities of the peaks of the (9,6) and (10,4) tubes increase rapidly during the first 510 min of annealing and they are not saturated after the last annealing step (2046 min). The (11,2) tube grows faster during the first 510 min and the peak intensity reaches the saturated value after 2046 min. The intensity of the peak of the (9,3) tube increases quickly during 254 min of annealing and becomes saturated after 510 min. For the (9,2) tube, there is a significant change in the peak intensity during 126 min and the value is almost saturated after 254 min (Figure 10a). At 560 °C, all nanotubes grow faster. The intensity of the (8,8) tube peak increases rapidly during the first 510 min and saturates after 2046 min. The (9,6), (10,4) and (11,2) tubes grow quickly during 254 min and the peak intensities reach almost saturated values after 1022 min. The peaks of the (9,3) and (9,2) tubes increase significantly during the first 126 and 62 min, respectively, and their intensities become saturated after 254 min (Figure 10b). With a subsequent increase in annealing temperature, the period of time in which rapid nanotube growth occurs and the annealing duration required to reach the saturated values of the peak intensities are reduced. At 580 °C, the intensities of the peaks of the (8,8), (9,6), (10,4) and (11,2) tubes are saturated after 1022 min of annealing, the (9,3) tube—after 254 min and the (9,2) tube—after 126 min (Figure 10c). At 600 °C, the intensities of the peaks of the (8,8), (9,6) and (11,2) tubes reach maximal values after 510 min, the (10,4) tube—after 254 min, the (9,3) tube—after 126 min and the (9,2) tube—after 62 min (Figure 10d). At 620 °C, the intensities of the peaks of the (8,8), (9,6), (10,4) and (11,2) tubes are almost saturated after 254 min, the (9,3) and (9,2) tubes—after 62 min (Figure 10e). At 640 °C, the time equals 254 min for the (8,8), (9,6), (10,4) and (11,2) tubes and 30 min—for the (9,3) and (9,2) tubes (Figure 10f).

Therefore, the growth rates of all inner nanotubes increase significantly with the increase in annealing temperature of the CoCp_2_-filled SWCNTs. Additionally, the growth rates of the inner tubes increase with the decrease in tube diameter: they are minimal for the largest-diameter (8,8) nanotube and maximal for the smallest-diameter (9,3) and (9,2) tubes. These trends are similar to the ones that were revealed for the NiCp_2_-filled SWCNTs.

While comparing the growth curves of the inner tubes inside the NiCp_2_- and CoCp_2_-filled SWCNTs (Figure 9 and Figure 10), one can notice the increased relative intensity of the peak of the (11,2) tube as compared to the peaks of the larger diameter (8,8), (9,6) and (10,4) tubes in the case of the CoCp_2_-filled SWCNTs. This effect probably has the same nature as the increased yield of the smaller-diameter (13,1) tube as compared to the (12,3) tube, which was observed in the Raman spectra of the annealed CoCp_2_-filled tubes acquired at a laser wavelength of 633 nm, discussed above. It is caused by the smaller size of CoCp_2_ as compared to NiCp_2_ molecules, which leads to the increased density of packing of the molecules inside the smaller diameter outer SWCNT and therefore larger yield of the smaller-diameter inner tube.

### 3.3. Growth Model and Calculation of Growth Rates of Inner Tubes

The observed growth dynamics of inner nanotubes evidently does not follow a simple exponential model that was reported for the catalyst lifetime-limited SWCNT growth in the CVD process. After an initial burst, the growth of inner tubes continues for tens of hours. In contrast, in the CVD synthesis, the growth of SWCNTs usually stops after tens of minutes due to the deactivation of the catalyst (the encapsulation of the catalyst particle by a passivating carbon layer). The key difference between the inner tube growth and the CVD growth is the well-shielded inner space of the host SWCNTs where the formation of inner tubes takes place in an undisturbed manner and the growth continues until no more carbon is available.

**Figure 11:**
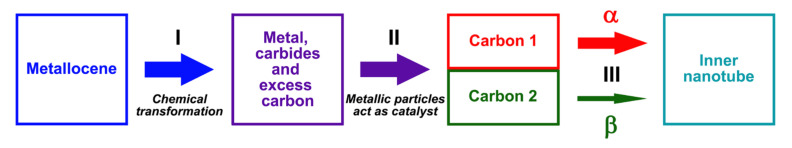
The illustration of the growth model of inner tubes from metallocene molecules encapsulated inside SWCNTs. The thermal treatment of the filled SWCNTs leads to the chemical transformation of metallocene to metal, metal carbides and excess carbon (step I). Metallic nanoparticles act as a catalyst and transform carbon to inner tubes (step II). The part of excess carbon (1) is processed rapidly at the beginning and forms the inner tube at a rate α. The other part of carbon (2) is transformed slower into the inner tube at a lower rate β (step III).

Nickelocene molecules are quickly decomposed and are known to form metastable nickel carbides at temperatures as low as 250 °C, which decompose to metallic nickel at temperatures higher than 450 °C [57]. As in the case of nickelocene, the cobaltocene molecules are decomposed upon annealing at low temperatures with the formation of metastable metal carbide surrounded by excess carbon, which is further decomposed to pure metal at temperatures higher than 500 °C [58,59,60]. The stoichiometry of 1:10 (M:C) in NiCp_2_ and CoCp_2_ molecules indicates a major excess of carbon. The thermal treatment of the metallocene-filled SWCNTs leads to the formation of the mixture of metal, metal carbides and excess carbon inside the nanotubes. These encapsulated substances are the precursors for inner tube growth. The following growth model can be proposed to explain the observed growth dynamics of inner tubes. Metallic nanoparticles surrounded by excess carbon inside the SWCNT channels act as catalysts for the inner tubes. A particle consumes carbon from one side and extrudes an inner nanotube at the other side. The part of excess carbon is processed rapidly at the beginning and forms the inner tube at a rate α. The other part of carbon is transformed slower into the inner tube at a lower rate β. Figure 11 illustrates the suggested growth model of inner tubes.

In this growth model, α determines the growth rate of inner nanotubes at the beginning, while β is observable after longer annealing hours. The first order linear differential equation may be written as in Equation (1). The signs are chosen in a way that meaningful, and α and β are always positive. The amount of carbon that is transformed to inner tubes is given by *A*. A part χ of this carbon is processed at the growth rate α and the other part (1−χ) of the carbon is processed at the growth rate β (0 ≤ χ ≤ 1). The amount of carbon in the form of grown inner nanotubes is given by *C*:

(1) $$\frac{dA}{d\tau }=A\left(\chi \alpha \;+\left(1-\chi \right)\beta \right)$$

(2) $$\frac{dC}{d\tau }=-\frac{dA}{d\tau }$$

The solution of the system may be readily found by an exponential ansatz:

C(τ)=A(1 −χe^−ατ^ − (1 −χ)e^−βτ^). (3)

This growth model fits the experimental growth curves of inner tubes well (Figure 7, Figure 8, Figure 9 and Figure 10). The fitting directly gives the values of the growth rates α and β. The results of the quantitative analysis of the growth curves confirm the above-discussed qualitative tendencies. The growth rates increase by tens of times (up to 100) with increasing annealing temperature. For example, for the (12,3) tube, the growth rate α increases from 0.0046 ± 0.0010 to 0.2640 ± 0.0880 min^−1^ (by the factor of 57) and the growth rate β increases from 0.0006 ± 0.0018 to 0.0179 ± 0.0063 min^−1^ (by the factor of 30) while increasing temperature from 520 to 600 °C. The (8,8), (12,3), (13,1) and (9,6) tubes grow very slowly at 480 and 500 °C and the (10,4) and (11,2) tubes—at 480 °C, so that their growth rates could not be quantified. The growth rates rise by tens of times (up to 30) with the decrease in tube diameter. For example, at 540 °C, the growth rates α and β increase from 0.0096 ± 0.0013 and 0.0016 ± 0.0028 min^−1^ for the (8,8) tube to 0.2786 ± 0.0597 and 0.0201 ± 0.0158 min^−1^ for the (9,2) tube (by the factors of 29 and 13, respectively). For all nanotubes, the growth rate α is larger than the growth rate β by several times (16, on the average). The calculated values of growth rates are consistent with the proposed growth dynamics of inner tubes.

The growth rates increase by tens of times (up to 100) with an increase in annealing temperature for cabaltocene. For example, for the (9,6) tube, the growth rate α increases from 0.0037 ± 0.0010 to 0.1691 ± 0.0082 min^−1^ (by the factor of 46) and the growth rate β increases from 0.0003 ± 0.0002 to 0.0036 ± 0.0005 min^−1^ (by the factor of 12) while temperature increases from 540 to 640 °C. The growth rates rise by tens of times (up to 39) with a decrease in tube diameter. For example, at 580 °C, the growth rates α and β increase from 0.0103 ± 0.0033 and 0.0016 ± 0.0009 min^−1^ for the (8,8) tube to 0.3310 ± 0.1356 and 0.0244 ± 0.0083 min^−1^ for the (9,2) tube (by the factors of 32 and 15, respectively). For all nanotubes, the growth rate α is larger than the growth rate β by several times (24, on the average). These tendencies are analogous to the ones that were observed for the nickelocene-filled SWCNTs.

### 3.4. Dependence of Growth Rates of Inner Tubes on Their Diameter and Annealing Temperature

**Figure 12:**
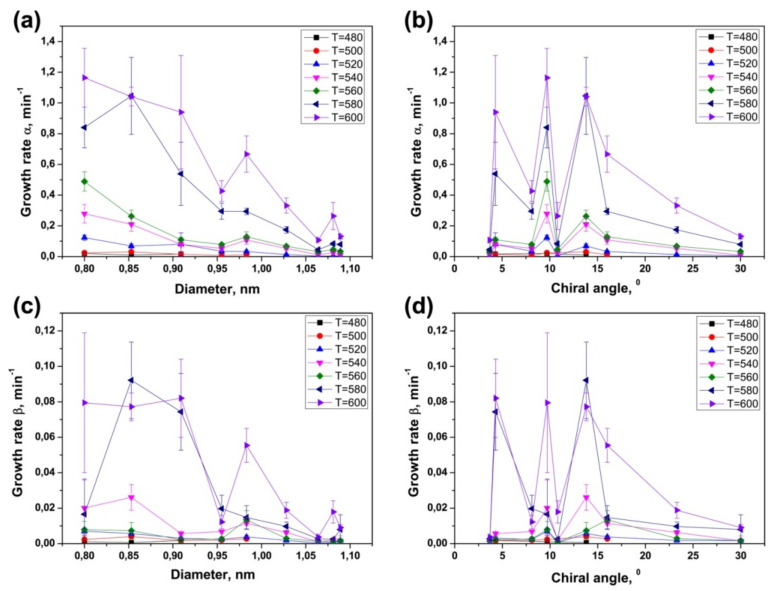
Growth rates α and β versus tube diameter (**a** and **c**, respectively) and versus chiral angle (**b** and **d**, respectively) for inner tubes with chiral vectors of (8,8), (12,3), (13,1), (9,6), (10,4), (11,2), (11,1), (9,3) and (9,2) formed upon *in situ* annealing of the nickelocene-filled SWCNTs at temperatures in a range from 480 to 600 °C.

**Figure 13:**
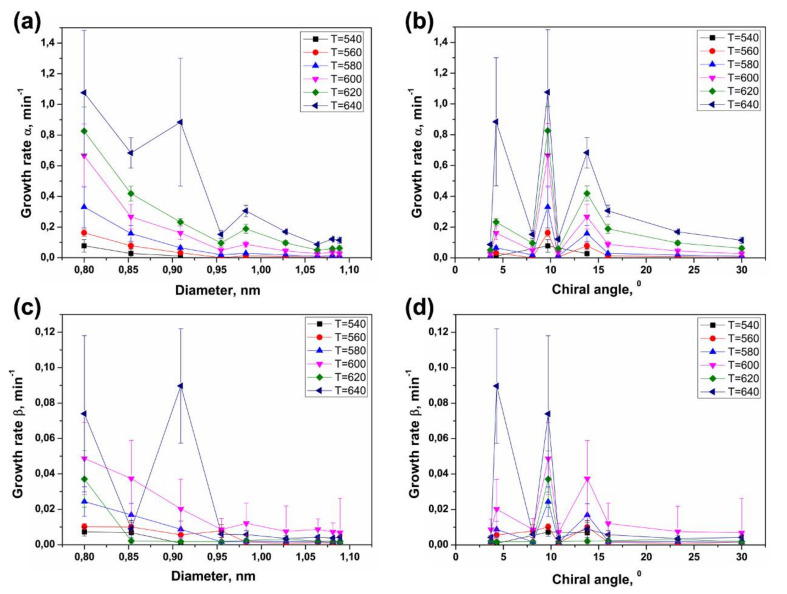
Growth rates α and β versus tube diameter (**a** and **c**, respectively) and versus chiral angle (**b** and **d**, respectively) for inner tubes with chiral vectors of (8,8), (12,3), (13,1), (9,6), (10,4), (11,2), (11,1), (9,3) and (9,2) formed upon *in situ* annealing of the cobaltocene-filled SWCNTs at temperatures in a range from 540 to 640 °C.

The data on the calculated growth rates (α and β) of various inner tubes at different annealing temperatures of the NiCp_2_- and CoCp_2_-filled SWCNTs are summarized in Figure 12 and Figure 13. These diagrams clearly show two trends: both growth rates of inner tubes increase nonlinearly (i) with decreasing the tube diameter at all temperatures and (ii) with increasing annealing temperature.

Studies on the CVD growth of nanotubes showed that their diameter was defined by the size of catalytic particles [61,62,63,64,65,66,67]. It was reported that reducing the size of the catalyst particles increased the growth rate of carbon filaments and nanotubes, independently on the type of catalyst [61,62,68,69,70,71], and that smaller-diameter nanotubes had higher growth rates than larger-diameter ones [61,62]. This effect was explained by the increased catalytic activity of smaller-diameter particles due to their larger specific surface area, larger curvature of surface and, consequently, larger amount of active sites [72,73] as well as the modified electronic structure [72], increased carbon solubility [69] and shortened diffusion length of carbon atoms to arrive at the growth site [62].

In the case of the inner tube growth, the catalytic particle is confined inside the outer SWCNTs that control its size. Thus, the diameter of growing inner tube is defined by the diameter of the outer nanotube. The difference in the diameter of nanotubes is given by the doubled van der Waals distance between the outer and inner graphene layers of DWCNTs (0.67 nm). The observed nonlinear increase in the growth rates of inner tubes with the decrease in their diameter can be attributed to the increase in the catalytic activity of smaller-diameter metallic nanoparticles, by analogy to the CVD process. The calculated growth rates of nanotubes in the CVD process were found to be inversely proportional to the tube diameter [61,63]. The experimental data on the inner tube growth fits well the dependence 1/*d_t_*. The growth rates increase by tens of times (up to 30) within the diameter change of less than 0.3 nm (Figure 12 and Figure 13).

It should be noted that although the correlation between the nanotube growth rate and its chiral angle was reported in the CVD synthesis of SWCNTs [21,22], the present results do not show a noticeable dependence of the growth rates of inner tubes on their chirality that goes beyond the cross-correlation of diameters and chiral angles.

Figure 12 and Figure 13 demonstrate a nonlinear increase in the growth rates α and β for all inner nanotubes with increasing annealing temperature. The same trend was observed for the CVD growth of carbon filaments [68,74,75,76] and nanotubes [17,18,19,20,23,63,65,66,69,77,78,79,80,81,82,83,84,85,86,87,88,89,90,91,92,93,94,95,96,97,98,99,100,101,102,103]. This effect is caused by the fact that the catalytic nanotube growth is a thermally activated process. Therefore, according to the Arrhenius equation, shown in Equation (4),

(4) $$\gamma =Be^{-\frac{E_{a}}{k_{B}T}}$$

where γ is the growth rate of nanotubes, *E_a_* is the activation energy of the nanotube growth, *k_B_* is the Boltzmann constant, *T* is the absolute temperature and *B* is a proportionality coefficient; the growth rate of nanotubes increases exponentially with temperature. It should be noted that deviations from this dependence observed for the growth rate β of several inner tubes grown inside the CoCp_2_-filled SWCNTs at the highest temperatures−620 and 640 °C (Figure 13) are probably caused by the fact that it is more difficult to achieve a fine temperature control at such high temperatures for long annealing durations.

### 3.5. Calculation of Activation Energies of the Inner Tube Growth

Taking into consideration the Arrhenius equation, shown in Equation (4), and its form after taking the natural logarithm (5),

(5) $$\ln\gamma =-\frac{E_{a}}{k_{B}T}+\ln B,$$

the activation energies of the inner tube growth were calculated from the linear fits of the dependence ln(growth rate) on 1/T. The slope of this linear dependence is $-\frac{E_{a}}{k_{B}}$. Thus, the linear fitting directly yields the value of the activation energy. This method for the calculation of activation energies is conventional and was used in many works dedicated to the investigation of the CVD growth of carbon filaments and nanotubes.

**Figure 14:**
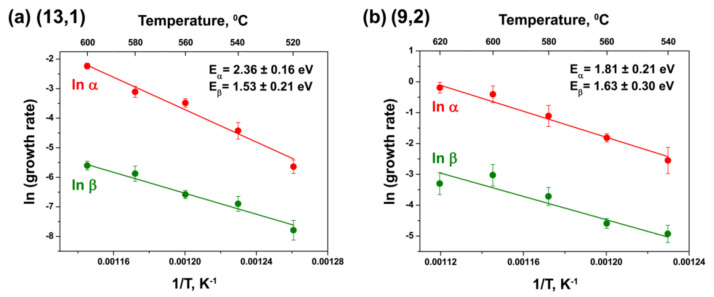
The plots of natural logarithms of the growth rates α and β versus inverse annealing temperature for the inner tubes with chiralities of (13,1) (**a**) and (9,2) (**b**) formed upon *in situ* annealing of the NiCp_2_− and CoCp_2_−filled SWCNTs, respectively. The top abscissa axis shows the annealing temperature in Celsius degrees for convenience. The calculated activation energies E_α_ and E_β_ are indicated together with their errors.

Figure 14 demonstrates the examples of the plots of natural logarithms of the growth rates α and β versus inverse annealing temperature for the (13,1) inner tube grown inside the NiCp_2_-filled SWCNTs and the (9,2) tube grown inside the CoCp_2_-filled SWCNTs. Every point in these plots corresponds to a data bar from Figure 12 and Figure 13. It should be noted that the growth rates of large-diameter inner tubes at the lowest temperatures and small-diameter tubes at the highest temperatures are not included in these plots, because the nanotubes grew very slowly or rapidly, respectively, and the growth rates had large errors.

The errors of the growth rates were recalculated to the logarithmic scale according to thefollowing formula:

(6) $$\xi =\ln(1+\frac{\delta \alpha }{\alpha }),$$

where δα is the error of the growth rate α and ξ is the recalculated error on the logarithmic scale.

All plots show linear dependences ln(growth rate) on 1/T. They are perfectly fitted with a linear function (Figure 14). The slopes of the linear fits give the values of two activation energies of the growth E_α_ and E_β_ for each inner nanotube. They are summarized in Table 2.

According to the obtained data, in the case of the NiCp_2_-filled annealed samples, the calculated activation energies E_α_range from 1.85 eV (for the (11,1) tube) to 2.57 eV (for the (8,8) and (12,3) tubes). The activation energies E_β_ have significantly smaller values than E_α_: they range from 1.49 eV (for the (10,4) and (11,2) tubes) to 1.91 eV (for the (9,3) tube). In the case of the CoCp_2_-filled annealed samples, the activation energies E_α_ range from 1.80 eV (for the (9,3) tube) to 2.71 eV (for the (10,4) tube). E_β_ values are noticeably smaller than E_α_: they range from 0.77 eV (for the (13,1) tube) to 1.79 eV (for the (11,1) tube) (Table 2).

### 3.6. Dependence of Activation Energies of the Inner Tube Growth on Their Diameter and Chirality

**Figure 15:**
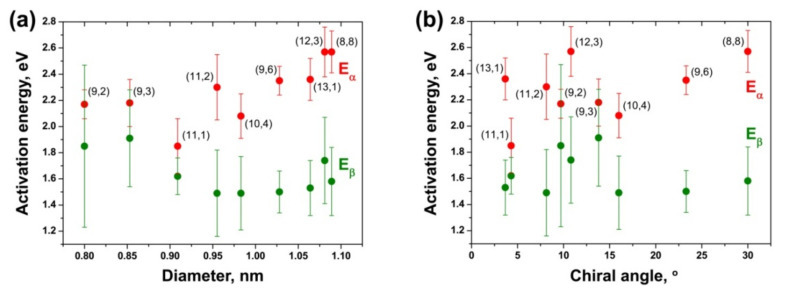
The activation energies E_α_ and E_β_ of the growth of the inner tubes with the chiralities of (8,8), (12,3), (13,1), (9,6), (10,4), (11,2), (11,1), (9,3) and (9,2) upon *in situ* annealing of the nickelocene-filled SWCNTs plotted versus the tube diameter (**a**) and chiral angle (**b**). The chiralities of inner tubes are denoted near the corresponding circles.

To compare the activation energies of the growth for different inner tubes, E_α_ and E_β_ were plotted versus the tube diameter and chiral angle. The obtained dependences for the NiCp_2_-filled annealed samples are presented in Figure 15. The plots of the activation energies versus the tube diameter (Figure 15a) demonstrate that E_α_ is smaller for the smaller-diameter inner nanotubes. The value gradually decreases as the tube diameter decreases. It drops by ~0.6 eV within the diameter change of less than 0.3 nm. The activation energy E_β_, which has smaller values than E_α_ by ~0.3–1.1 eV, shows no dependence on the tube diameter for the largest-diameter inner tubes and an increase by ~0.4 eV for the smallest-diameter tubes. However, the values of the smallest-diameter tubes have the largest errors, too.

The plots of the activation energies versus the tube chiral angle (Figure 15b) demonstrate that E_α_ and E_β_ do not depend on the chirality of inner tubes. A slight decrease in the values with decreasing chiral angles that is observed for E_α_ is connected to the diameter-dependence of the activation energy. It is caused by the cross-correlation of diameters and chiral angles for the considered inner tubes (the chiral angles are larger for the largest-diameter inner tubes).

**Figure 16:**
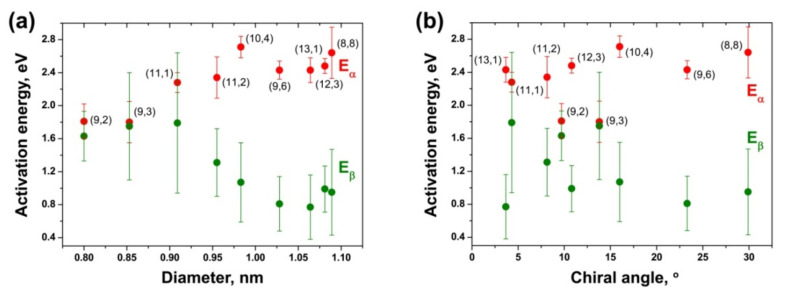
The activation energies E_α_ and E_β_ of the growth of the inner tubes with the chiralities of (8,8), (12,3), (13,1), (9,6), (10,4), (11,2), (11,1), (9,3) and (9,2) upon *in situ* annealing of the cobaltocene-filled SWCNTs plotted versus the tube diameter (**a**) and chiral angle (**b**). The chiralities of inner tubes are denoted near the corresponding circles.

Figure 16 demonstrates the plots of the activation energies E_α_ and E_β_ of different inner nanotubes versus the tube diameter and chiral angle for the CoCp_2_-filled annealed samples. The dependence of the activation energies on the tube diameter (Figure 16a) shows a step-by step decrease in E_α_ with a decrease in the tube diameter. It drops by ~0.8 eV while the tube diameter decreases from ~1.1 to 0.8 nm. Therefore, the observed diameter dependence of E_α_ is slightly more prominent than the one revealed for the nickelocene-filled nanotubes (Figure 16a). The activation energy E_β_ demonstrates dependence on the tube diameter, too. For the largest-diameter nanotubes, E_β_ has smaller values than E_α_ by up to ~1.8 eV. As the tube diameter decreases, E_β_ increases gradually by up to ~1.0 eV and reaches the value of E_α_ for the smallest-diameter tubes. Despite the fact that the activation energies of these tubes have the largest errors, the observed diameter dependence of E_β_ is clear and it is more prominent than in the case of the NiCp_2_-filled nanotubes, where an increase in E_β_as the tube diameter decreases was within the error range of the calculated activation energies (Figure 16a).

The plots of the activation energies E_α_ and E_β_ versus the tube chiral angle (Figure 16b) show that they do not depend on the chirality of inner tubes, as in the case of NiCp_2_-filled SWCNTs (Figure 15b).

### 3.7. Discussion of the Growth Process of Inner Tubes

To summarize, the data on the growth of inner tubes with chiralities of (8,8), (12,3), (13,1), (9,6), (10,4), (11,2), (11,1), (9,3) and (9,2) upon *in situ* annealing of the nickelocene- and cobaltocene-filled SWCNTs, presented in the previous sections, show that the growth kinetics of nanotubes are characterized by two growth rates,α and β. They correspond to two rates of the transformation of carbon from decomposed nickelocene into nanotubes on catalytic metallic nanoparticles. At the beginning of annealing, a quick transformation with a α rate is observed, whereas at longer annealing times, a slower transformation with a β rate occurs. At different annealing temperatures, β is smaller than α by an average of ~20 times. The growth rates α and β differ for various inner tubes. Both growth rates increase as the tube diameter decreases and are independent of the tube chiral angle. At different annealing temperatures, α and β increase by up to 40 times within the tube diameter variation of less than 0.3 nm (between 0.800 and 1.089 nm). Two activation energies E_α_ and E_β_ correspond to the inner tube growth with rates α and β. In the case of the NiCp_2_-filled annealed samples, the calculated values of E_α_ and E_β_ vary for different inner tubes and range from 1.85 to 2.57 eV and from 1.49 to 1.91 eV, respectively. E_α_ decreases gradually as the tube diameter decreases, whereas E_β_ shows no significant dependence on the tube diameter. Both activation energies of the inner tube growth do not show a noticeable dependence on chirality. In the case of the CoCp_2_-filled annealed samples, the calculated values of E_α_ and E_β_ range from 1.80 to 2.71 eV and from 0.77 to 1.79 eV, respectively. E_α_ and E_β_ show the opposite dependence on the inner tube diameter. E_α_ decreases gradually as the tube diameter decreases, whereas E_β_ increases and reaches the value of E_α_ for the smallest-diameter tubes. Both activation energies do not depend on the chirality of inner tubes.

The significant difference between the values of two activation energies E_α_ and E_β_ that was revealed may testify that the growth rate-limiting mechanism changes during the growth of inner nanotubes. The calculated values of activation energies E_α_ and E_β_ for the NiCp_2_- and CoCp_2_-filled annealed samples fit well into the range of reported energies for solid-state carbon diffusion through bulk metals and metal carbides. Indeed, the activation energy of the bulk diffusion of carbon in nickel with a face-centered cubic crystal lattice was measured to be 1.43–1.74 eV [104,105,106,107,108]. The activation energy of the bulk diffusion of carbon in cobalt with a hexagonal closely packed crystal lattice was measured to be 1.50–1.68 eV [104,109,110]. There are no reports on the calculation of the activation energy of carbon diffusion in nickel and cobalt carbides, possibly due to their metastability. However, it is known that the activation barrier of carbon diffusion in metal carbides is higher than in the corresponding metals [72]. The increased activation energy of carbon diffusion in metal carbides as compared to pure metals was explained by the fact that the diffusion of carbon in metal carbides is no longer interstitial, as in the case of low carbon concentrations in metals, but it is mediated by thermal vacancies in the metal and carbon sub-lattices [72,111].

Thus, the calculated activation energies E_α_ and E_β_ for the NiCp_2_- and CoCp_2_-filled annealed samples can be assigned to energies of solid-state carbon diffusion through metal carbide and pure metal, respectively. Therefore, these values show that catalyst particles are in a solid state during inner tube growth. Although the melting point of metallic nanoparticles can be significantly reduced by the size effects and carbon incorporation [72], the growth temperatures of inner tubes (480–600 °C) are too low to melt catalyst nanoparticles.

The calculated activation energies E_α_ and E_β_ show that metal carbide is formed in the beginning of the growth process of inner tubes, and it is transformed to pure metal later. Nickel carbides are known to be metastable [58] and decompose at temperatures higher than 400 °C [112,113,114]. Recent X-ray photoelectron spectroscopy (XPS) studies on the inner tube growth upon the annealing of the nickelocene-filled SWCNTs showed that metastable nickel carbides were formed at temperatures as low as 250 °C and decomposed to metallic nickel at temperatures higher than 450 °C [57]. Cobalt carbide is known to decompose at temperatures higher than 500 °C [58,59,60]. This means that metallic particles act as catalysts of inner tube growth.

Taking into consideration the above-discussed literature data and calculated activation energies of the inner tube growth, it can be concluded that Ni and Co catalyst nanoparticles undergo carburization in the beginning of the growth process, with the subsequent decomposition of metal carbide to pure metal. The formation of metastable metal carbide is only possible when a large amount of excess carbon is available around metal nanoparticles. This is the case at short annealing times, and it leads to a fast growth of inner tubes with a rate α. At longer annealing times, most of nearby excess carbon is used off; its amount is not enough to restore decomposed metal carbide and the inner nanotubes grow with a slower rate β.

**Figure 17:**
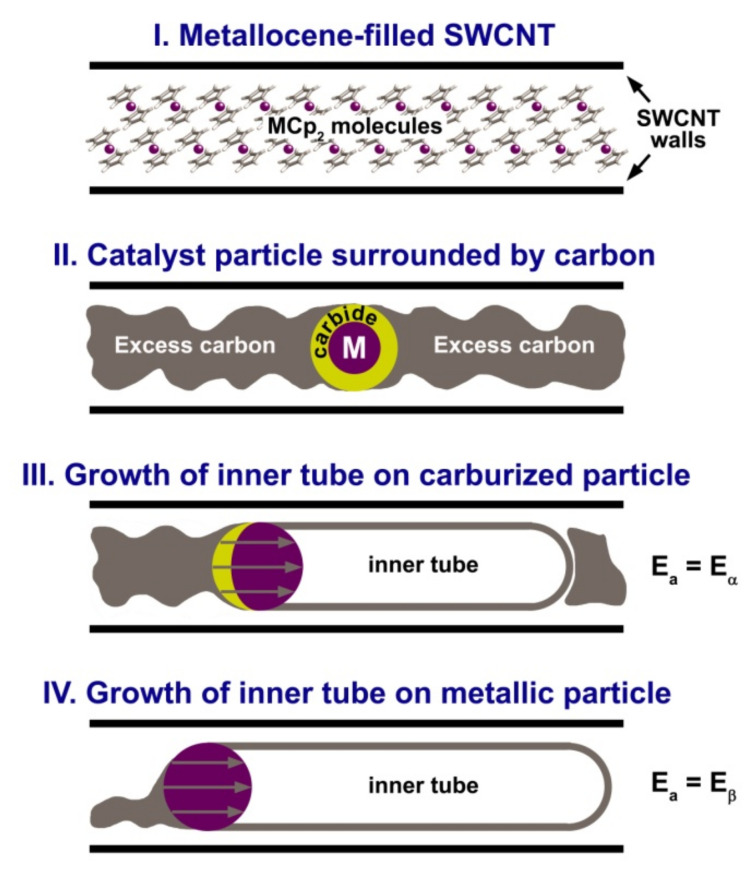
Illustration of the growth model of the inner tubes inside the metallocene-filled SWCNTs. MCp_2_ (M = Ni, Co) molecules encapsulated into the channel of SWCNT (step I) are decomposed upon annealing. As a result, carburized metal nanoparticles surrounded by excess carbon are formed (step II). They act as a catalyst for the inner tube growth (step III). Carbon diffuses from one side to other side of the catalyst particle through its bulk (as shown by arrows), and the inner nanotube grows with the activation energy *E_a_* = E_α_. When the amount of excess carbon becomes insufficient to restore the decomposing metastable metal carbide, the growth of the inner tube continues on the metallic catalyst nanoparticle (step IV). Carbon diffuses through the bulk of the particle (as shown by arrows), and the nanotube is formed with the activation energy *E_a_* = E_β_. The growth stops when there is no more carbon available.

Thus, the following growth model of inner tubes inside the nickelocene- and cobaltocene-filled SWCNTs can be proposed on the basis of the obtained experimental data. It is illustrated in Figure 17. The annealing of the NiCp_2_- and CoCp_2_-filled SWCNTs leads to the decomposition of metallocene with the formation of metal nanoparticles surrounded by a major excess of carbon inside the SWCNT channels, because of the stoichiometry of 1:10 (M:C) in the NiCp_2_ and CoCp_2_ molecules. Metallic nanoparticles confined in between walls of SWCNTs act as catalysts of the inner tube growth. The catalyst nanoparticle dissolves nearby carbon, and it leads to the formation of metastable intermediate metal carbide. In the above-described reports, the formation of intermediate carbide was observed in the bulk or in the subsurface layer of the catalyst particle. Taking into consideration a small size of the catalyst particle (~1 nm) in our case, it can be assumed that the metal nanoparticle is mostly carburized, although its core may remain as pure metal. The growth of the inner tube occurs as a result of the diffusion of carbon from one side to other side of the catalyst particle through its bulk. It is driven by a large carbon concentration gradient between the side of the particle that is contiguous with the excess carbon and the side where the nanotube grows. The bulk diffusion of carbon is the growth rate-limiting process and therefore at this stage, the activation energy of the inner tube growth (E_α_) is related to the activation energy of the bulk solid-state diffusion through metal carbide. As a metastable phase, metal carbide exists as long as there is enough excess carbon to restore the decomposing compound. When the amount of carbon becomes insufficient, metal carbide decomposes to pure metal. The growth of the inner tube continues in the metallic catalyst nanoparticle. At this stage, the activation energy of the nanotube growth (E_β_) is related to the activation energy of bulk solid-state diffusion through metal. The growth of the inner tube stops when there is no more carbon available.

A gradual decrease in E_α_ as the inner tube diameter decreases is probably caused by the fact that the structure of metal carbide becomes more defective with a decrease in the size of the catalyst nanoparticle. This is connected to the increased specific surface area and amount of surface states for smaller nanoparticles. As was mentioned above, carbon diffusion in carbides is not interstitial, but it is mediated by thermal vacancies in the metal and carbon sub-lattices. Consequently, a more defective structure leads to a smaller energy barrier for the diffusion of carbon through the bulk of metal carbide. In the case of the metal catalyst, an increase in the defectiveness of the structure with a decrease in the size of nanoparticle is less prominent, because there is only one type of atom in the lattice. Carbon diffusion in metal is interstitial and the change in the defectiveness of the particle structure influences the activation barrier of the diffusion to a lesser extent than for metal carbide. Because the activation energies E_α_ and E_β_ are related to the activation barrier of carbon diffusion through the catalyst nanoparticle, they are the property of the particle and do not depend on the chirality of the grown inner nanotube.

To summarize, *in situ* Raman spectroscopy allowed for the time-dependent inner tube growth inside nickelocene- and cobaltocene-filled SWCNTs to be investigated with a time resolution of several minutes. In contrast to conventional methods of nanotube synthesis, the outer SWCNTs provided a well-shielded environment for the catalyst nanoparticles that formed as a product of the decomposition of metallocene, and the growth of the inner tubes took place in an undisturbed manner. Functional catalytic metallic nanoparticles were tightly confined inside the channels of SWCNTs and thus featured two separated sides, one for processing the carbon feedstock and the other for growing the nanotubes. This led to the complete elimination of the otherwise unavoidable lifetime-limiting process of catalyst poisoning, in which the whole particle would be encapsulated by a passivating carbon layer. As a result, the growth of inner tubes continued for tens of hours and only stopped when there was no more carbon feedstock available. The outer SWCNTs defined the size of the catalytic nanoparticles and therefore the diameter of the inner tubes. Precise control of the nanotube diameter allowed the growth process of individual-chirality inner tubes to be monitored and their growth rates and activation energies to be determined. Two different regimes of growth kinetics of the inner tubes were revealed. They were connected with different chemical compositions of the catalyst nanoparticles, namely pure metal or metal carbide. The measured activation energies showed that the activation barrier of the nanotube growth depended on the size of the catalyst nanoparticle. The activation energies were found to increase as the size of metal carbide-containing catalyst particles increased and to decrease as the size of metallic catalyst particles increased.

To the best of our knowledge, only two previous reports were dedicated to the investigation of the growth kinetics and the determination of the growth rates of individual-chirality nanotubes [21,22]. The present work pioneers the measurement of the activation energies for the growth of individual-chirality tubes and provides the first evidence of the size-effect dependence of the activation energy. This work makes a large step towards a better understanding of the growth dynamics of individual-chirality nanotubes. It also proves that it is feasible to tailor catalyst/support systems that can grow nanotubes for tens of hours. With a steady carbon feedstock, such catalyst/support systems can be envisaged for the continuous production of ultra-long nanotubes.

### 3.8. Comparison of Activation Energies of the Inner Tube Growth inside the NiCp_2_- and CoCp_2_-Filled SWCNTs

**Figure 18:**
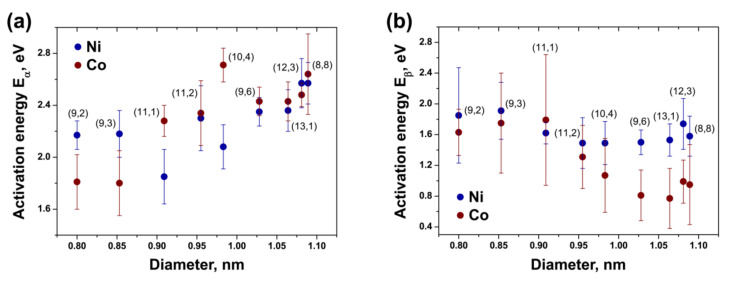
The comparison of the activation energies E_α_ (**a**) and E_β_ (**b**) of the growth of the inner tubes with the chiralities of (8,8), (12,3), (13,1), (9,6), (10,4), (11,2), (11,1), (9,3) and (9,2) upon *in situ* annealing of the nickelocene- and cobaltocene-filled SWCNTs plotted versus the tube diameter. The chiralities of inner tubes are denoted near the corresponding points.

Although the growth of inner nanotubes inside NiCp_2_- and CoCp_2_-filled SWCNTs occurs *via* the same mechanism, and the values of activation barriers of the bulk solid-state carbon diffusion through nickel and cobalt are very similar, the activation energies E_α_ and E_β_ of the tube growth show differences for the nickel and cobalt catalysts. Figure 18 compares the plots of the activation energies E_α_ and E_β_ versus the inner tube diameter for both catalysts. E_α_ decreases gradually as the tube diameter decreases and demonstrates no noticeable dependence on the catalyst nature (Figure 18a). A decrease in E_α_ as the tube diameter decreases is connected with a higher defectiveness of the structure of the metal carbide layer for smaller catalyst nanoparticles due to the increased amount of surface states. Because carbon diffusion in carbides is not interstitial, but it is mediated by thermal vacancies in the metal and carbon sub-lattices, the higher defectiveness of the structure results in a smaller energy barrier for the diffusion of carbon through the bulk of metal carbide. The irregular structure of the carbide layer of the catalyst particles and carbon diffusion through vacancies apply equally well to Ni and Co. There are indeed no noticeable differences between the values of the activation energy E_α_ of the inner tube growth on the nickel and cobalt catalysts.

In contrast, the activation energy E_β_ and its dependence on the tube diameter (Figure 18b) differ significantly for the two catalysts. In the case of the nickel catalyst, only a slight increase in E_β_ as the tube diameter decreases is observed, which is within the error range of the calculated energies. In the case of the cobalt catalyst, the diameter dependence of E_β_ is much more prominent, because the values of the large-diameter inner tubes (*d_t_* = ~0.95–1.10 nm) are noticeably reduced, whereas the values of the small-diameter tubes (*d_t_* = ~0.80–0.95 nm) are similar to the ones observed for the nickel catalyst. The decrease in the activation energy E_β_ for the large-diameter inner tubes in the case of cobalt may be related to its different bulk crystal structure and coordination preferences as compared to nickel. Metallic cobalt has a hexagonal closely packed crystal structure with a longer c-axis in the unit cell. Nickel has a face-centered cubic crystal lattice with a cubic unit cell. Therefore, being confined within the SWCNT channel, the cobalt catalyst particle of ~1 nm size will have a more disordered structure with a larger number of interstitial sites and, consequently, smaller energy barriers for the bulk carbon diffusion. An increase in the activation energy E_β_ for the small-diameter inner tubes and its equalization for both metals may testify to the formation of similar well-ordered structures for small-diameter nickel and cobalt catalyst nanoparticles, which have smaller numbers of interstitial sites and, consequently, increased energy barriers for bulk carbon diffusion.

Indeed, structural transitions of encapsulated metals depending on the diameter of host nanotubes were reported previously [115,116,117,118]. The authors of [115] observed the formation of various types of Gd nanowires inside nanotubes with different diameters *via* HRTEM. Inside the large-diameter tubes (1.8 and 5 nm), the nanowires with bulk crystal-like structures were formed. In contrast, inside the small-diameter tubes (0.64 nm), the nanowires with a unique chain-like structure that is specific to the 1D nanotube space were obtained. Similarly, using HRTEM and DFT calculations, the authors of [117] found that small cross-section Mo wires with unique 1D structures were formed inside DWCNTs with the inner tube diameters between 0.7 and 1.0 nm. For zigzag (*n*,0) tubes, this diameter range corresponds to *n* = 9,...,14. No nanowires were observed within the tubes with a diameter less than 0.7 nm (*n* < 9). Inside the tubes with a diameter more than 1.0 nm (*n* > 14), the nanowires with bulk crystal-like structures were formed. These results suggest that the structure of encapsulated nanowires becomes more and more similar to the bulk-like crystal structure as the nanowire diameter increases. This is consistent with previous studies on metal-filled large-diameter SWCNTs and MWCNTs, which showed that the structure of nanowires was identical to the corresponding bulk crystal structure [119,120,121,122,123]. Inside small-diameter tubes, the formation of metallic nanowires with unique 1D atomic structures was reported [116,118,124,125].

Thus, the observed dependence of E_β_ on the inner tube diameter and catalyst type is caused by the fact that large metallic catalyst particles possess structures that have a reminiscence to their respective bulk crystal structures, whereas small metallic catalyst particles possess structures that are completely defined by the outer SWCNT (with no signatures of bulk properties). The critical diameter of the catalyst particle is ~0.95 nm, which is in agreement with the above-discussed literature data. The different bulk structures of nickel and cobalt cause differences in the activation energy E_β_ of the growth of the large-diameter tubes (*d_t_* = ~0.95–1.10 nm). For the small-diameter tubes (*d_t_* = ~0.80–0.95 nm), E_β_ is similar for two metals, because Ni and Co have very close atomic radii (0.124 and 0.125 nm, respectively [58]).

It should be noted that the revealed significant differences in the dependences of E_α_ and E_β_ on the tube diameter and catalyst nature may be additional evidence that only part (a subsurface layer) of the metallic catalyst nanoparticle is carburized during the inner tube growth. An irregularity of the structure of the carbide layer for both metals increases as the particle size decreases, and it leads to the observed diameter-dependence and no metaldependence of E_α_. In the case of the purely metallic catalyst nanoparticle, the metal type and structure of the particle define the value of the activation energy E_β_.

It is worth noticing that in the case of the nickel and cobalt catalysts, no dependence of the activation energies E_α_ and E_β_ on the chirality of the inner tubes is observed (Figure 15b and Figure 16b). This is caused by the fact that E_α_ and E_β_ correspond to the energy barrier of carbon diffusion through the catalyst nanoparticle, and therefore they are a property of the particle and not related to the chirality of the growing nanotube.

**Figure 19:**
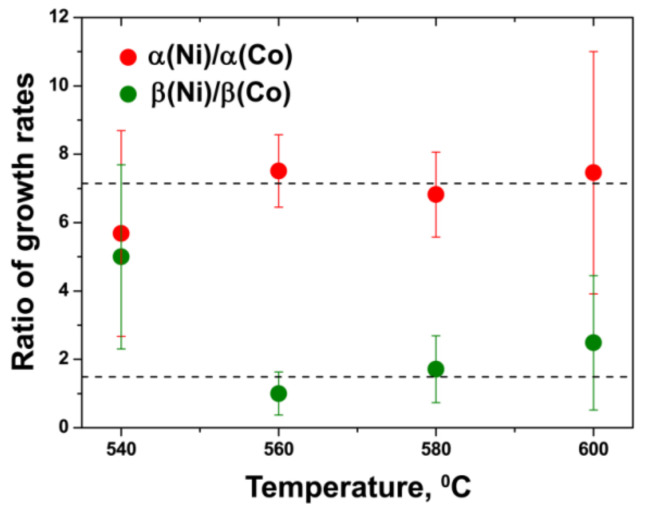
The ratios of the rates α (red) and β (green) of the growth of the (12,3) inner tube inside the nickelocene- and cobaltocene-filled SWCNTs plotted *versus* annealing temperature. The dashed horizontal lines denote the average values of the ratios of the growth rates for different annealing temperatures.

A noticeable difference between the inner tube growth process inside the nickelocene and cobaltocene-filled SWCNTs is the range of growth temperatures of nanotubes. The temperatures of the inner tube growth are noticeably higher in the case of CoCp_2_ (540–640 °C) than NiCp_2_ (480–600 °C). At the same temperature, the growth rates α and β of all inner tubes are significantly larger for NiCp_2_. Figure 19 presents the ratios of the rates of the growth of the mean-diameter (12,3) inner tube inside the NiCp_2_- and CoCp_2_-filled SWCNTs plotted versus annealing temperatures, which overlap for two metallocenes. The values of the ratios of α and β are approximately constant at different annealing temperatures. The average values of the ratios amount to 7.1 for α and 1.5 for β, as denoted by the dashed horizontal lines in Figure 19. This means that the growth of the inner tube on the carburized Ni nanoparticle occurs with a 7.1-times higher rate than on the carburized Co particle. The growth of the nanotube on the metallic Ni nanoparticle occurs with a 1.5-times higher rate than on the metallic Co particle.

The dependence of the growth rates of nanotubes on the type of catalyst in the CVD process was investigated experimentally and theoretically [64,69,78,82,88,126]. However, contradictory results were reported, which is possibly connected with different synthesis processes and, therefore, nanotube growth mechanisms. The first group of authors demonstrated that among iron-group metals (Ni, Co, Fe), Ni catalyst yielded the highest growth rate of nanotubes in the PECVD [64] and thermal CVD process using ferrocene and nickelocene as the catalyst source and C_2_H_2_ as the carbon source [69]. These results were in line with theoretical calculations [126]. The second group of authors reported that the Fe catalyst led to the highest growth rate of nanotubes in the thermal CVD synthesis using C_2_H_2_ as the carbon source [78] and in the pyrolysis of metal phthalocyanines [82]. The third group of authors showed that Ni and Co catalysts had a similar effectiveness in the PECVD synthesis of nanotubes, which was higher than for Fe catalyst [88]. The observed differences were explained by different solubility and diffusion constants of carbon in metals and rates of integration of carbon into growing tubes [127,128,129,130,131,132,133].

Indeed, in the growth process that is controlled by bulk carbon diffusion through the metallic particle, such as the inner tube growth process under study, the growth rate of tubes is governed by the diffusion rate of carbon. The growth rate can be defined by the formula:

*v* = *k_d_C_c_*, (7)

where *v* is the growth rate of tubes, *k_d_* is proportional to the diffusion coefficient of carbon in bulk metal and *C_c_* is the saturated concentration of carbon in bulk metal [78,82]. The diffusion coefficient is known to change inversely with the carbon solubility in a metal and to depend strongly on the carbon concentration [72]. The authors of Ref. [78] took into consideration the diffusion coefficients of carbon in bulk nickel and cobalt (9.5 × 10^−8^ and 4.4 × 10^−8^ cm^2^ s^−1^ at 900 °C, and 2.9 × 10^−7^ and 1.5 × 10^−7^ cm^2^ s^−1^ at 1000 °C, respectively) and saturated concentrations of carbon (0.2–0.3 wt% in the temperature range of 900–1000 °C for both metals) and estimated the relative ratio of the growth rates of MWCNTs on nickel and cobalt catalysts to be 2:1 at these temperatures. This is in good agreement with the ratio of 1.5 obtained in the present work for growth rate β of the (12,3) inner tube on the metallic Ni and Co nanoparticles (Figure 19). The inner tubes grow at sufficiently lower temperatures; however, the diffusion coefficient and saturated concentration of carbon change in the same way with temperature for the two metals. Therefore, these results additionally prove that the bulk solid-state diffusion of carbon through the metallic catalyst particle is the rate β-determining step in the inner tube growth inside the NiCp_2_- and CoCp_2_-filled SWCNTs, and the growth rate β is proportional to the diffusion coefficient and saturated concentration of carbon in bulk metal.

The ratio of the α rate of the growth of the (12,3) inner tube on carburized Ni and Co particles with a subsurface carbide layer is ~4.7 times higher than the ratio of the β rate of the growth on the metallic particles (Figure 19). This is probably caused by different types of carbon diffusion in metal carbides (through thermal vacancies in the metal and carbon sub-lattices) as compared to metals (through interstitial sites in the lattice). Nickel carbide is stable until lower temperatures (~450 °C) than cobalt carbide (~500 °C). A difference in the thermal stability of ~50 °C leads to the effect that at the same temperature, there are more vacancies in the structure of nickel carbide and they have a larger mobility than in cobalt carbide. This results in the increased rates of the bulk carbon diffusion through a subsurface carbide layer of Ni catalyst nanoparticles as compared to Co particles and, consequently, larger growth rates α of the inner tubes.

## 4. Conclusions

To summarize, individual-chirality selective *in situ* Raman spectroscopy studies on time-dependent inner tube growth inside NiCp_2_- and CoCp_2_-filled SWCNTs in the time range between several minutes up to tens of hours allowed conclusive information on growth dynamics to be derived in a well-shielded environment. The decomposition of metallocene formed long-lived metallic catalyst nanoparticles tightly confined inside the SWCNT channels. They had two distinct active sides for consuming the carbon feedstock and growing the nanotube, which prevented them from encapsulation by a passivating carbon shell. There were two successive stages of the nanotube growth on the carburized and purely metallic catalyst nanoparticles, respectively. These stages were characterized by the corresponding growth rates and activation energies. They were found to depend on the diameter of the catalyst particles. Both growth rates increased inversely proportionally for smaller diameter particles due to the increased catalytic activity. The activation energies of the nanotube growth on the carburized metallic particles decreased monotonically as the diameter decreased, because of the more defective structure of the metal carbide layer with a larger amount of surface states. In contrast, the activation energies of the tube growth on the metallic particles increased for smaller-diameter particles due to a transition from bulk-like to a 1D structure.

The identical growth mechanisms for inner tubes on the Ni and Co catalysts allowed the rates and activation energies to be compared quantitatively and the differences that are characteristic to the two metals to be elucidated. The growth temperatures of inner tubes were ~40 °C higher on the Co particles. At the same temperature, the growth rates of all inner tubes on the carburized and metallic Ni particles were significantly larger than on the Co particles. The difference in the growth rates on the carburized catalysts was related to the smaller thermal stability of nickel carbide, which resulted in a higher concentration of more mobile vacancies. The difference in the growth rates on the purely metallic catalysts was in line with the diffusion coefficients of carbon in the respective bulk metals. The activation energies of the tube growth on the carburized Ni and Co particles did not show differences, as the carbon diffusion through vacancies in either bulk carbide suggests. The activation energies of the tube growth on the metallic Ni and Co particles differed for the large-diameter tubes (*d_t_* = ~0.95–1.10 nm) because of their different bulk structures, and they were the same for the small-diameter tubes (*d_t_* = ~0.80–0.95 nm), where both metals with very close atomic radii underwent a transition to 1D packing.

In conclusion, the present work pioneers the calculation of the rates and activation energies of the growth of nine individual-chirality inner tubes inside NiCp_2_- and CoCp_2_-filled SWCNTs and provides a comprehensive picture of their growth dynamics. There are no reports in the literature on the quantitative comparison of the rates and activation energies of the growth of nanotubes on the Ni and Co catalysts in such precisely controlled conditions. In this work, the dependence of the rates and activation energies of the growth of individual-chirality tubes on the metal catalyst type is firstly revealed. The obtained results mark a large amount of progress in the understanding of the influence of the catalyst nature on the chirality-specific growth dynamics of nanotubes.

## Figures and Tables

**Table 1:** The positions of peaks of inner tubes in the RBM-band of Raman spectra of the NiCp_2_- and CoCp_2_-filled annealed samples acquired at laser wavelengths of 633 nm (E_ex_ = 1.96 eV) and 568 nm (E_ex_ = 2.18 eV) as well as chiralities, diameters, chiral angles and excited electronic transitions of inner tubes that were assigned to these peaks.

Laser Wavelength	RBM Peak Position (cm^−1^)	Inner Nanotube Chirality	Inner Nanotube Diameter (nm)	Inner Nanotube Chiral Angle (°)	Excited Electronic Transition
633 nm	214	(12,3)	1.081	10.8	$E_{11}^{M}$
	219	(13,1)	1.064	3.7	$E_{11}^{M}$
	254	(11,1)	0.909	4.3	$E_{22}^{S}$
568 nm	215	(8,8)	1.089	30.0	$E_{11}^{M}$
	226	(9,6)	1.028	23.3	$E_{11}^{M}$
	235	(10,4)	0.983	16.0	$E_{11}^{M}$
	240	(11,2)	0.955	8.1	$E_{11}^{M}$
	269	(9,3)	0.853	13.8	$E_{11}^{M}$
	285	(9,2)	0.800	9.7	$E_{22}^{S}$

**Table 2:** The calculated activation energies E_α_ and E_β_ (together with their errors) of the growth of the (8,8), (12,3), (13,1), (9,6), (10,4), (11,2), (11,1), (9,3) and (9,2) inner nanotubes upon the *in situ* annealing of the NiCp_2_- and CoCp_2_-filled SWCNTs.

Inner Nanotube Chirality	Activation Energy (eV)			
	Precursor			
	NiCp_2_		CoCp_2_	
	E_α_	E_β_	E_α_	E_β_
(8,8)	2.57 ± 0.16	1.58 ± 0.26	2.64 ± 0.31	0.95 ± 0.52
(12,3)	2.57 ± 0.19	1.74 ± 0.33	2.48 ± 0.09	0.99 ± 0.28
(13,1)	2.36 ± 0.16	1.53 ± 0.21	2.43 ± 0.15	0.77 ± 0.39
(9,6)	2.35 ± 0.11	1.50 ± 0.16	2.43 ± 0.11	0.81 ± 0.33
(10,4)	2.08 ± 0.17	1.49 ± 0.28	2.71 ± 0.13	1.07 ± 0.48
(11,2)	2.30 ± 0.25	1.49 ± 0.33	2.34 ± 0.25	1.31 ± 0.41
(11,1)	1.85 ± 0.21	1.62 ± 0.14	2.28 ± 0.12	1.79 ± 0.85
(9,3)	2.18 ± 0.18	1.91 ± 0.37	1.80 ± 0.25	1.75 ± 0.65
(9,2)	2.17 ± 0.11	1.85 ± 0.62	1.81 ± 0.21	1.63 ± 0.30

## Data Availability

The data presented in this study are available on request from the corresponding author.
